# Reduced inhibition, bursting, and accelerated oscillations drive early hippocampal hyperactivity in Alzheimer’s disease in vivo

**DOI:** 10.1038/s42003-026-09918-y

**Published:** 2026-03-28

**Authors:** Soraya Meftah, Sungmin Kang, Mingshan Liu, Xingran Wang, Ada Nursel Topçu, Maialen Martin Abad, Áron Kőszeghy, Long Wan, Conor Mullin, Lida Zoupi, Jian Gan

**Affiliations:** 1 UK Dementia Research Institute at the University of Edinburgh, Edinburgh, UK; 2 Institute for Neuroscience and Cardiovascular Research, the University of Edinburgh, Edinburgh, UK; 3 Simons Initiative for Developing Brain, the University of Edinburgh, Edinburgh, UK

**Keywords:** Diseases of the nervous system, Hippocampus, Neurophysiology

## Abstract

‘Early hippocampal hyperactivity’ is well-documented in Alzheimer’s disease (AD), yet its mechanisms in vivo before amyloid-β plaque deposition at the levels of synaptic transmission and neural oscillations remain unclear. Here, we perform in vivo high-resolution patch-clamp and high-throughput probe recordings in the hippocampus of amyloidopathy mice before plaque deposition. At the cellular level, we observe reduced inhibitory synaptic input, hypoactivity of fast-spiking interneurons, and enhanced bursting in pyramidal neurons. At the network level, we reveal accelerated hippocampal oscillations, characterized by increased theta and beta power. Mechanistically, this acceleration stems from selectively strengthened synchrony of oscillation-associated excitatory synaptic currents at higher frequencies. Our findings provide in vivo evidence linking early hippocampal hyperactivity to specific alterations in synaptic transmission dynamics and network dysfunction, highlighting that accelerated oscillations in the hippocampus could be a functional biomarker for early AD and a therapeutic target for restoring network stability before cognitive decline occurs.

## Abstract (web summary)

Early hippocampal hyperactivity in AD before Aβ plaque deposition could be explained in vivo by reduced inhibitory synaptic transmission with enhanced pyramidal cell bursting and accelerated neural oscillations driven by increased theta/beta rhythms.

## Introduction

Alzheimer’s disease (AD) is a devastating neurodegenerative condition that profoundly impairs cognitive function and quality of life, imposing significant burdens on individuals, families, and society^1^. Pathologically, AD is characterized by the gradual accumulation of amyloid-beta (Aβ) proteins, transitioning from an early stage of soluble oligomers and fibrils to a late stage of diffuse and compact plaques, alongside intra- and extracellular tau aggregates^2–4^. Whilst Aβ plaques have been a focus of late-stage therapeutic intervention, increasing evidence highlights early Aβ pathology, e.g., the toxic role of soluble Aβ in early AD pathophysiology in vivo^5–7^, particularly in the hippocampus^8,9^, with studies suggesting that this toxicity may be reversible^8–10^. Consequently, early detection and intervention for AD are increasingly viewed as both critical and feasible.

Beyond blood and cerebrospinal fluid biomarkers, functional markers aimed at detecting early alterations in neural activity have been investigated, primarily through electroencephalogram (EEG) recordings of neocortical activity, positron emission tomography (PET), and functional magnetic resonance imaging (fMRI) for deeper brain structures^11^. However, technical limitations, particularly in the spatial and temporal resolution, have hindered the exploration of neural activity in deep brain regions such as the hippocampus. The hippocampus plays a pivotal role in memory and spatial cognition—functions that are severely disrupted in AD—and is among the earliest brain regions affected by the disease, even before the clinical onset of dementia^12–14^. Thus, identifying a quantifiable marker of early hippocampal dysfunction would hold diagnostic and therapeutic value.

A promising pre-symptomatic functional marker is early hippocampal hyperactivity^15^. In human studies, this phenomenon has been observed as an enhanced blood oxygen level-dependent (BOLD) signal by fMRI in patients with mild cognitive impairment (MCI)^16,17^, pre-symptomatic familial AD^18^, and carriers of high-risk AD genes, such as *APOE4*, before clinical AD symptoms^19–22^. Moreover, reducing this early hyperactivity in the hippocampus pharmacologically improved cognitive performance in patients^17^, highlighting its clinical relevance. Animal models have provided some cellular mechanistic insight into hippocampal hyperactivity/hyperexcitability at points with high Aβ plaque pathology. Higher burst firing in pyramidal cells linked to dendritic structure^23^, changes in neuronal synchrony linked to altered population entropy^24^, and enhanced excitation/inhibition ratio due to reduced inhibitory inputs^25^ are just some of these identified mechanisms. For early Aβ pathology, in vivo studies of amyloidopathy mouse models reported increased susceptibility to seizures and network hypersynchrony in the hippocampus prior to plaque accumulation^26–29^. Furthermore, an increased proportion of hyperactive hippocampal neurons via two-photon Ca^2+^ imaging was reported when Aβ is still largely soluble^8^, or upon direct application of Aβ oligomers^9^. Rescue of GABAergic signaling might be one promising therapeutic target, with evidence of efficacy shown in mouse models of amyloidopathy^30–32^. However, the exact relationship between synaptic, cellular, and network mechanisms in vivo in response to increased Aβ in early amyloid pathology in the hippocampus has yet to be fully elucidated.

Therefore, in this study, we set out to uncover the cellular and network mechanisms that underpin ‘early hippocampal hyperactivity’ by examining hippocampal oscillations and the underlying synaptic mechanisms in vivo in a mouse model of amyloidopathy prior to the onset of typical Aβ plaque-associated pathology. Employing in vivo patch-clamp and simultaneous local field potential (LFP) recordings in young (2–3 months) APP_swe_/PS1ΔE9 (henceforth APP/PS1) mice^33^, we demonstrated a reduction of basal synaptic inhibition, contributing to overall imbalanced excitation/inhibition inputs. In addition, enhanced phase-locking of synaptic excitation to theta but not delta oscillations underlies accelerated hippocampal oscillations. Furthermore, using high-throughput silicon probe recordings in young (3–5 months) awake animals, we observed similar accelerated hippocampal oscillations characterized by increased beta power. At the cellular level, pyramidal cells exhibited stronger burst firing of action potentials, whereas fast-spiking interneurons showed hypoactivity, as evidenced by increased proportion of low-firing-rate population, consistent with reduced synaptic inhibition revealed by in vivo patch-clamp recordings. In summary, our findings provide direct in vivo evidence for mechanisms underpinning hippocampal hyperactivity in early amyloidopathy at cellular and functional network levels. We also identify a potential quantifiable functional marker of abnormal neural oscillations in the hippocampus that may aid in early diagnosis.

## Results

### Synaptic inhibition is reduced in the hippocampus in vivo in anesthetized young APP/PS1 mice

To investigate synaptic mechanisms underlying early hippocampal hyperactivity in AD, we utilized the well-established APP/PS1 mouse model. Our study focused on a young cohort (2–3 months old), at an age prior to typical Aβ plaque pathology, when large numbers of Aβ plaques are not yet formed^34,35^ (also see Supplementary Fig. 1). APP/PS1 animals and their age-matched WT controls were included, with both male and female mice represented.

**Fig. 1:**
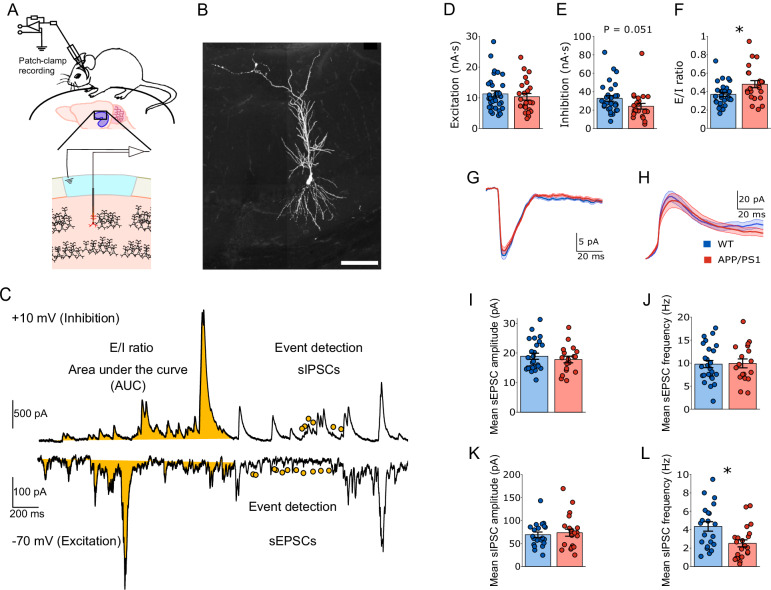
Synaptic excitation and inhibition in young anesthetized APP/PS1 mice in vivo. **A** Schematic diagram showing in vivo whole-cell patch-clamp recording from the CA1 region of the hippocampus. **B** Representative image of a recorded CA1 pyramidal neuron following in vivo whole-cell patch-clamp recording. Scale bar: 50 µm. **C** Example voltage-clamp traces of spontaneous excitatory (sEPSCs, bottom, −70 mV) and inhibitory (sIPSCs, top, +10 mV) postsynaptic currents. Total charge transfer (area under the curve, AUC, in yellow) was quantified for excitatory and inhibitory currents, respectively. Filled dots in yellow indicate quantified spontaneous excitatory or inhibitory events using the algorithm described in the methods section. **D**–**F** Overall strength of synaptic excitation, inhibition, and E/I ratio. **D** No significant difference in total charge transfer was observed between WT (blue) and APP/PS1 (red) mice for excitation. **E** APP/PS1 mice exhibited a trend of reduction in total inhibitory strength compared to WT mice (*P* = 0.051). **F** E/I ratio was significantly elevated in APP/PS1 mice (*P* = 0.015). Data are presented as mean ± SEM in bar graphs with individual values in dots. Averaged synaptic currents at −70 mV for excitation (sEPSCs) (**G**) and +10 mV for inhibition (sIPSCs) (**H**). Thick line represents the mean, and shading represents SEM. **I**–**L** Synaptic transmission properties. **I** Mean sEPSC amplitude was comparable between genotypes. **J** Mean sEPSC frequency did not differ significantly between groups. **K** Mean sIPSC amplitude was unchanged. **L** Mean sIPSC frequency was significantly reduced in APP/PS1 mice compared to WT (*P* = 0.019), indicating a presynaptic mechanism underlying reduced inhibitory transmission. Data are presented as mean ± SEM in bar graphs with individual values in dots. Asterisks indicate statistical significance (*P < 0.05).

We conducted in vivo whole-cell patch-clamp recordings from hippocampal CA1 pyramidal cells under ketamine/xylazine general anesthesia (Fig. 1A, B). Voltage-clamp configuration was employed to precisely measure excitatory and inhibitory synaptic inputs within the same neuron, respectively. Spontaneous EPSCs (sEPSCs) and IPSCs (sIPSCs) were recorded to assess synaptic activity (Fig.1C).

First, to evaluate the overall strength of synaptic excitation and inhibition, we quantified the total charge transfer (area under the curve). No significant differences in excitatory input strength were observed between genotypes (Fig. 1D; 10.335 ± 1.079 nA·s for APP/PS1, *n* = 24 (14 animals); 11.281 ± 1.004 nA·s for WT, *n* = 32 (18 animals); *P* = 0.520 *F*_(1,54)_ = 0.419). However, a trend of reduction in inhibitory input strength reached the border of statistical significance in APP/PS1 mice (Fig. 1E; 24.156 ± 3.108 nA·s for APP/PS1, *n* = 24 (14 animals); 32.776 ± 3.073 nA·s for WT, *n* = 29 (18 animals); *P* = 0.051, *F*_(1,51)_ = 3.979). The excitation/inhibition (E/I) ratio exhibited a significant elevation, likely due to reduced synaptic inhibition (Fig. 1F; 0.480 ± 0.039 for APP/PS1, *n* = 23 (14 animals); 0.369 ± 0.023 for WT, *n* = 29 (18 animals); *P* = 0.015, *F*_(1,50)_ = 6.394).

Next, to investigate potential pre- or postsynaptic mechanisms underlying these changes, we examined the frequency and amplitude of baseline sEPSCs and sIPSCs, respectively. No significant differences were observed in sEPSC amplitude (Fig. 1I; 17.801 ± 1.101pA for APP/PS1, *n* = 18 (12 animals); 18.898 ± 1.017 pA for WT, *n* = 26 (17 animals); *P* = 0.467, *F*_(1,42)_ = 0.540) or sEPSC frequency (Fig. 1J; 9.949 ± 0.967 Hz for APP/PS1, *n* = 18 (12 animals); 9.795 ± 0.787 Hz for WT, *n* = 26 (17 animals); *P* = 0.947, *F*_(1,42)_ = 0.005). However, in APP/PS1 mice, a significant reduction in sIPSC frequency (Fig. 1L; 2.517 ± 0.379 Hz for APP/PS1, *n* = 22 (13 animals); 4.353 ± 0.516 Hz for WT, *n* = 21 (15 animals); *P* = 0.019, *F*_(1,41)_ = 5.982) was detected, whereas sIPSC amplitude remained unchanged (Fig. 1K; 73.381 ± 7.639 pA for APP/PS1, *n* = 22 (13 animals); 69.028 ± 5.764 pA for WT, *n* = 21 (15 animals); *P* = 0.646, *F*_(1,41)_ = 0.214). Overall, these results suggested a presynaptic mechanism could be responsible for the overall reduction of inhibitory transmission, leading to an increased E/I ratio.

**Fig. 2:**
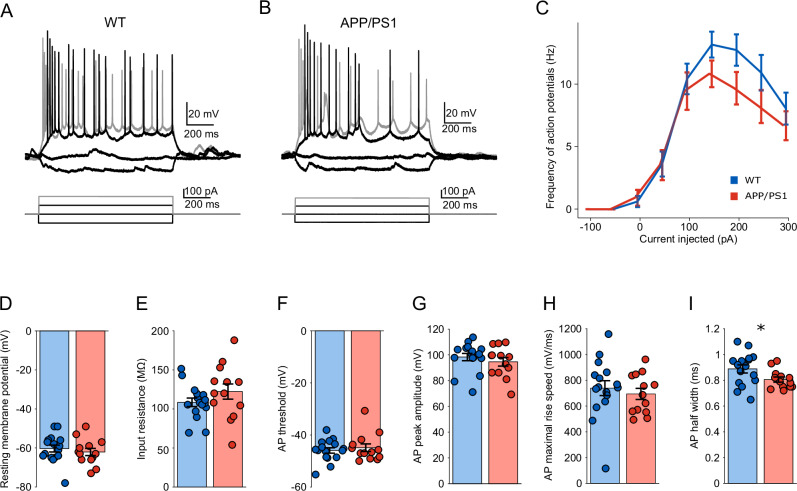
Intrinsic properties of CA1 pyramidal neurons in young anesthetized APP/PS1 mice in vivo. Representative in vivo current-clamp recording traces from CA1 pyramidal neurons in WT (**A**) and APP/PS1 (**B**) mice in response to injected stepwise current injections. **C** Input-frequency relationship showing action potential frequency as a function of injected current. No significant difference was detected between genotypes. **D**–**I** Quantification of intrinsic membrane and action potential properties. **D** Resting membrane potential was comparable between groups. **E** Input resistance did not differ significantly between genotypes. **F** The action potential threshold remained unchanged. **G** Action potential peak amplitude was similar between WT and APP/PS1 mice. **H** The maximum action potential rise speed was not significantly different. **I** Action potential half-width was decreased in APP/PS1 mice compared to WT (*P* = 0.049), suggesting altered spike kinetics. Data are presented as mean ± SEM in bar graphs with individual values in dots. Asterisks indicate statistical significance (*P < 0.05).

To assess potential changes in the intrinsic properties of pyramidal neurons in the CA1 region, we conducted current-clamp recordings in a separate set of CA1 pyramidal cells in vivo (Fig. 2A, B). No significant differences were observed in key passive membrane properties, including resting membrane potential (Fig. 2D, −62.077 ± 1.848 mV for APP/PS1, *n* = 13 (11 animals); −60.313 ± 1.784 mV for WT, *n* = 16 (15 animals); *P* = 0.575, *F*_(1,27)_ = 0.322) and input resistance (Fig. 2E, 122.231 ± 9.713 MΩ for APP/PS1, *n* = 13 (11 animals); 108.500 ± 5.435 MΩ for WT, *n* = 16 (15 animals); *P* = 0.185, *F*_(1,27)_ = 1.848). Similarly, no overt change in input-frequency firing patterns was detected (Fig. 2C, *P* = 0.140, *F*_(1,248)_ = 2.193, *n* = 13 (11 animals) for APP/PS1, *n* = 16 (15 animals) for WT, genotype sweeps interaction). Furthermore, action potential threshold (Fig. 2F, −44.885 ± 1.468 mV for APP/PS1, *n* = 13 (11 animals); −45.956 ± 1.039 mV for WT, *n* = 16 (15 animals); *P* = 0.442, *F*_(1,27)_ = 0.610), peak amplitude (Fig. 2G, 94.600 ± 3.303 mV for APP/PS1, *n* = 13 (11 animals); 98.306 ± 2.820 mV for WT, *n* = 16 (15 animals); *P* = 0.382, *F*_(1,27)_ = 0.791), and maximal rise speed (Fig. 2H, 694.246 ± 43.006 mV·ms^−1^ for APP/PS1, *n* = 13 (11 animals); 739.244 ± 57.929 mV·ms^−1^ for WT, *n* = 16 (15 animals); *P* = 0.540, *F*_(1,27)_ = 0.385) remained indistinguishable between genotypes. Notably, AP half-width was reduced in APP/PS1 animals (Fig. 2I, 0.805 ± 0.020 ms for APP/PS1, *n* = 13 (11 animals); 0.888 ± 0.031 ms for WT, *n* = 16 (15 animals; *P* = 0.049, *F*_(1,27)_ = 4.236). Overall, these data suggested there was no overt change in intrinsic properties in CA1 pyramidal cells in young APP/PS1 mice in vivo.

### Neural oscillations are accelerated in the hippocampus in anesthetized young APP/PS1 mice in vivo

Next, we moved up our focus from single-cell physiology to neural network-level analysis. Neural oscillations are quantifiable measurements of brain function and directly underlie normal cognitive computation. We took advantage of our in vivo anesthetized preparation, which preserved the integrity of neural circuitry and the key oscillations involved in memory encoding, consolidation, and retrieval in the hippocampus, albeit with limitations inherent to the anesthetized condition. We sought to determine whether hippocampal oscillations are altered in the early stages of AD, where there are high levels of Aβ species, and if so, to identify the underlying synaptic mechanisms.

**Fig. 3:**
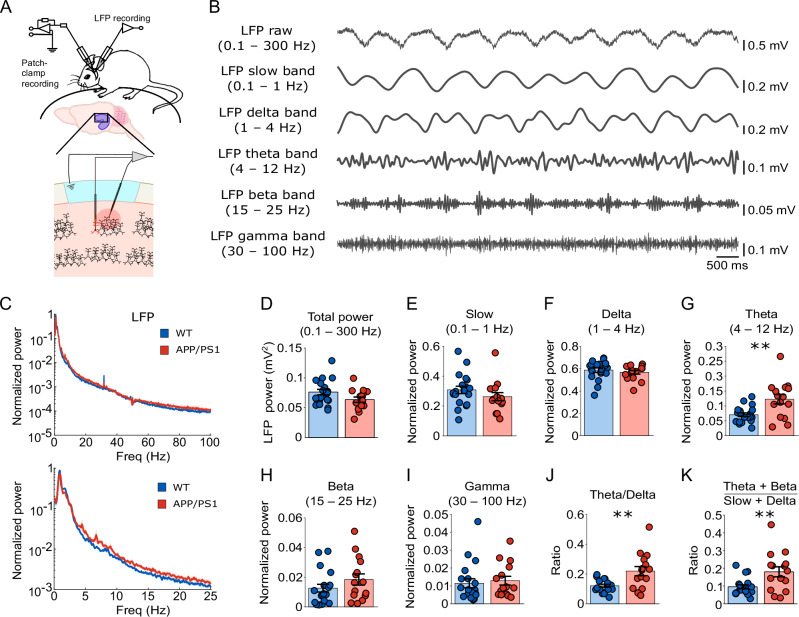
Altered hippocampal network oscillations in anesthetized young APP/PS1 mice. **A** Schematic diagram showing simultaneous in vivo voltage-clamp and LFP recordings from the CA1 region of the hippocampus. **B** Representative LFP traces in the slow (0.1–1 Hz), delta (1–4 Hz), theta (4–12 Hz), beta (15–25 Hz), and gamma bands (30–100 Hz). **C** Normalized LFP power spectra, 0.1–100 Hz (upper) and 0.1–25 Hz (lower), in WT (blue) and APP/PS1 (red) mice. Thick line represents the mean, and shading represents SEM. Quantification of total LFP power (**D**) and normalized power in slow wave (0.1–1 Hz, **E**), delta (1–4 Hz, **F**), theta (4–12 Hz, **G**), beta (15–25 Hz, **H**), and gamma (30–100 Hz, **I**) bands, respectively. Note a significant increase in the theta band (*P* = 0.005) in APP/PS1 mice. **J**, **K** APP/PS1 mice exhibited increased ratios of theta/delta (*P* = 0.004) and theta + beta/slow + delta (*P* = 0.009). Together with increased theta power in (**G**), these data indicate an acceleration of hippocampal oscillations. WT mice are shown in blue, APP/PS1 mice in red. Data are presented as mean ± SEM in bar graphs with individual values in dots. Asterisks indicate statistical significance (*P < 0.05, **P < 0.01).

To address this, we performed simultaneous LFP and voltage-clamp recordings in young APP/PS1 mice (Fig. 3A). No significant difference was observed in total LFP power between genotypes (0.1–300 Hz; Fig.3D, 0.064 ± 0.004 mV^2^ for APP/PS1, *n* = 15 animals; 0.076 ± 0.005 mV^2^ for WT, *n* = 19 animals; *P* = 0.063, *F*_(1,32)_ = 3.719). To account for the influence of the aperiodic component in the LFP, we computed the power spectrum of the periodic component by subtracting the aperiodic component from the raw LFP spectrum^36^ (see Methods). The magnitude of the aperiodic component was indistinguishable across genotypes (Supplementary Fig. 2A, D). The resulting aperiodic-subtracted power spectrum was used in the following analysis.

An analysis of the relative power (Fig.3C) revealed a significant increase in the theta band (4–12 Hz; Fig.3G, 0.122 ± 0.016 for APP/PS1, *n* = 15 animals; 0.070 ± 0.006 for WT, *n* = 19 animals; *P* = 0.005, *F*_(1,32)_ = 9.221) in APP/PS1 animals, with a significantly higher theta/delta ratio (Fig. 3J; 0.219 ± 0.031 for APP/PS1, *n* = 15 animals; 0.121 ± 0.010, *n* = 19 animals; *P* = 0.004, *F*_(1, 32)_ = 9.532). Consistently, the theta + beta to slow + delta ratio was elevated (Fig. 3K; 0.180 ± 0.028 for APP/PS1, *n* = 15 animals; 0.095 ± 0.011 for WT, *n* = 19; *P* = 0.009, *F*_(1, 32)_ = 7.788). However, the relative power of other frequency bands—including slow wave (0.1–1 Hz), delta (1–4 Hz), beta (15–25 Hz), and gamma (30–100 Hz)—remained comparable between genotypes (Fig. 3E, slow wave: 0.263 ± 0.027 for APP/PS1, 0.308 ± 0.025 for WT, *P* = 0.162, *F*_(1, 32)_ = 2.051; Fig.3F, delta: 0.568 ± 0.017 for APP/PS1, 0.587 ± 0.020 for WT, *P* = 0.237, *F*_(1, 32)_ = 1.450; Fig.3H, beta: 0.018 ± 0.004 for APP/PS1, 0.013 ± 0.003 for WT, *P* = 0.197, *F*_(1, 32)_ = 1.736; Fig.3I, gamma: 0.013 ± 0.002 for APP/PS1, 0.011 ± 0.003 for WT, *P* = 0.380, *F*_(1, 32)_ = 0.793); *n* = 15 animals for APP/PS1, *n* = 19 animals for WT). These results, characterized by an increased proportion of theta frequency component as well as an elevation of theta/delta ratio and theta + beta to slow + delta ratio in LFP, indicated an overall acceleration of hippocampal oscillations in young APP/PS1 mice. This interpretation was corroborated by a significant reduction of absolute power in slow wave and delta (Supplementary Fig. 3).

### Accelerated hippocampal oscillations associate with phase-locked excitatory transmission at higher oscillatory frequencies in anesthetized young APP/PS1 mice

LFPs predominantly reflect the summation of synaptic transmission arising from coordinated firings of large neuronal populations. To investigate the synaptic mechanisms underlying the observed acceleration of hippocampal oscillations, we examined oscillation-associated excitatory and inhibitory synaptic transmission that may contribute to the reduction of delta and the increase of theta power in LFPs in APP/PS1 mice.

**Fig. 4:**
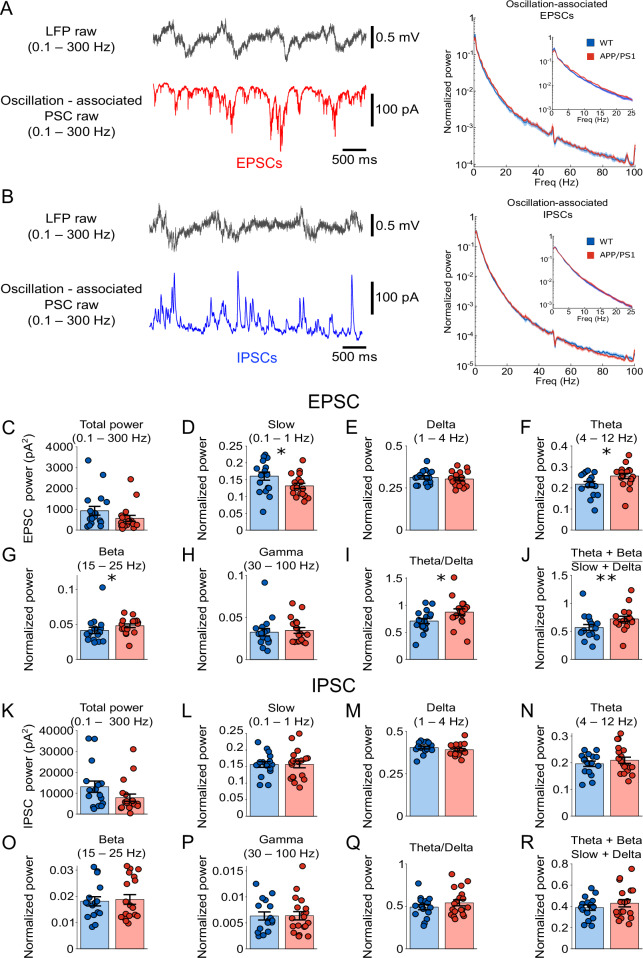
Altered oscillation-associated synaptic currents in anesthetized young APP/PS1 mice. **A** Left: Representative oscillation-associated excitatory postsynaptic currents (EPSCs) recorded in WT and APP/PS1 mice. Right: Normalized power spectral density of oscillation-associated EPSCs (0.1–100 Hz) in WT (blue) and APP/PS1 mice (red), respectively. Thick line represents the mean, and shading represents SEM. Insets show zoomed-in power spectrum (0.1–25 Hz). **B** Same as in (**A**), but for oscillation-associated inhibitory postsynaptic currents (IPSCs). Quantification of total EPSC power (**C**), and normalized EPSC power in slow wave (**D**), delta (**E**), theta (**F**), Beta (**G**), and gamma (**H**), respectively. APP/PS1 mice exhibited significantly reduced slow wave power (*P* = 0.038), but increased theta (*P* = 0.017) and beta power (*P* = 0.019), indicating a shift of oscillation-associated excitatory synaptic input towards faster dynamics. **I**, **J** Quantification of theta/delta and theta + beta/slow + delta ratio. APP/PS1 mice showed a significantly elevated theta/delta ratio (*P* = 0.028) and composite theta + beta/slow + delta ratio (*P* = 0.006). **K**–**R** Same analysis as in (**C**–**J**) but for oscillation-associated IPSCs. Data are presented as mean ± SEM in bar graphs with individual values in dots. Asterisks indicate statistical significance (*P < 0.05, **P < 0.01).

We first analyzed the power spectrum of oscillation-associated synaptic currents (Fig. 4A, B). In EPSCs, the total power remained largely unchanged (Fig.4C; 560.7737 ± 142.0379 pA^2^ for APP/PS1, *n* = 18 (12 animals); 925.4258 ± 213.1671 pA^2^ for WT, *n* = 17 (14 animals); *P* = 0.078, *F*_(1,33)_ = 3.320). When relative power was analyzed, APP/PS1 mice showed a significant reduction in the slow wave component (Fig. 4D; 0.132 ± 0.008 for APP/PS1, *n* = 18 (12 animals); 0.161 ± 0.012 for WT, *n* = 17 (14 animals); *P* = 0.038, *F*_(1,33)_ = 4.651) and significant increases in the theta (Fig. 4F; 0.257 ± 0.012 for APP/PS1, *n* = 18 (12 animals); 0.218 ± 0.013 for WT, *n* = 17 (14 animals); *P* = 0.017, *F*_(1,33)_ = 6.300) and beta bands (Fig. 4G; 0.048 ± 0.002 for APP/PS1, *n* = 18 (12 animals); 0.041 ± 0.005 for WT, *n* = 17 (14 animals); *P* = 0.019, *F*_(1,33)_ = 5.287). No significant genotype differences were observed in the delta (Fig. 4E; 0.303 ± 0.012 for APP/PS1; 0.313 ± 0.01 for WT; *P* = 0.683, *F*_(1,33)_ = 0.683) or gamma (Fig. 4H; 0.035 ± 0.004 for APP/PS1; 0.033 ± 0.004 for WT, *P* = 0.639, *F*_(1,33)_ = 0.224) bands. The theta/delta ratio (Fig. 4I; 0.874 ± 0.059 for APP/PS1; 0.709 ± 0.048 for WT; *P* = 0.028, *F*_(1,33)_ = 5.287) and the composite theta + beta to slow + delta ratio were significantly increased in APP/PS1 mice (Fig. 4J; 0.722 ± 0.048 for APP/PS1, *n* = 18 (12 animals); 0.570 ± 0.052 for WT, *n* = 17 (14 animals); *P* = 0.006, *F*_(1,33)_ = 8.474). These findings suggested that although the overall magnitude of oscillation-associated excitatory drive remained intact, its inner dynamics became faster in young APP/PS1 mice.

For oscillation-associated IPSCs, no significant difference in total power was observed in APP/PS1 animals (Fig.4K; 7866.800 ± 1737.300 pA^2^ for APP/PS1, *n* = 19 (12 animals); 13117.000 ± 2695.500 pA^2^ for WT, *n* = 16 (14 animals); *P* = 0.083, *F*_(1,33)_ = 3.205), and no change was observed in relative power across genotypes in slow wave (Fig.4L; 0.157 ± 0.010 for APP/PS1, *n* = 19 (12 animals); 0.157 ± 0.009 for WT, *n* = 16 (14 animals); *P* = 0.739, *F*_(1,35)_ = 0.113), delta (Fig.4M; 0.391 ± 0.008 for APP/PS1, *n* = 19 (12 animals); 0.405 ± 0.008 for WT, *n* = 16 (14 animals); *P* = 0.103, *F*_(1,33)_ = 2.809), theta (Fig.4N; 0.209 ± 0.012, *n* = 19 (12 animals); 0.196 ± 0.010 for WT, *n* = 16 (14 animals); *P* = 0.719, *F*_(1,33)_ = 0.132), beta (Fig.4O; 0.019 ± 0.002 for APP/PS1, *n* = 19 (12 animals); 0.018 ± 0.001, *n* = 16 (14 animals); *P* = 0.842, *F*_(1,33)_ = 0.041), or gamma (Fig.4P; 0.006 ± 0.001 for APP/PS1, *n* = 19 (12 animals); 0.006 ± 0.001 for WT, *n* = 16 (14 animals); *P* = 0.887, *F*_(1,33)_ = 0.020). Similarly, theta/delta ratio remained unchanged (Fig. 4Q; 0.541 ± 0.036 for APP/PS1, *n* = 19 (12 animals); 0.491 ± 0.031 for WT, *n* = 16 (14 animals); *P* = 0.598, *F*_(1,33)_ = 0.283), nor in theta + beta to slow + delta ratio (Fig. 4R; 0.428 ± 0.034 for APP/PS1, *n* = 19 (12 animals); 0.388 ± 0.026 for WT, *n* = 16 (14 animals); *P* = 0.618, *F*_(1,33)_ = 0.253). These findings suggested that the overall oscillation-associated inhibitory drive as well as its internal dynamics remained similar across genotypes.

Based on these findings, we further examined the precise temporal relationship between excitatory or inhibitory synaptic events with hippocampal oscillations in young APP/PS1 mice by performing a phase-locking analysis. We first detected individual EPSCs and IPSCs occurring in conjunction with hippocampal oscillations using a derivative-based event detection method^37,38^, and then analyzed their timing relative to oscillation cycles of delta (1–4 Hz) and theta (4–12 Hz) bands. To identify oscillation cycles for phase-locking analysis, delta and theta epochs were detected using a cycle-by-cycle, threshold-based method^39^ following parameterization^36^ (See Methods for details).

**Fig. 5:**
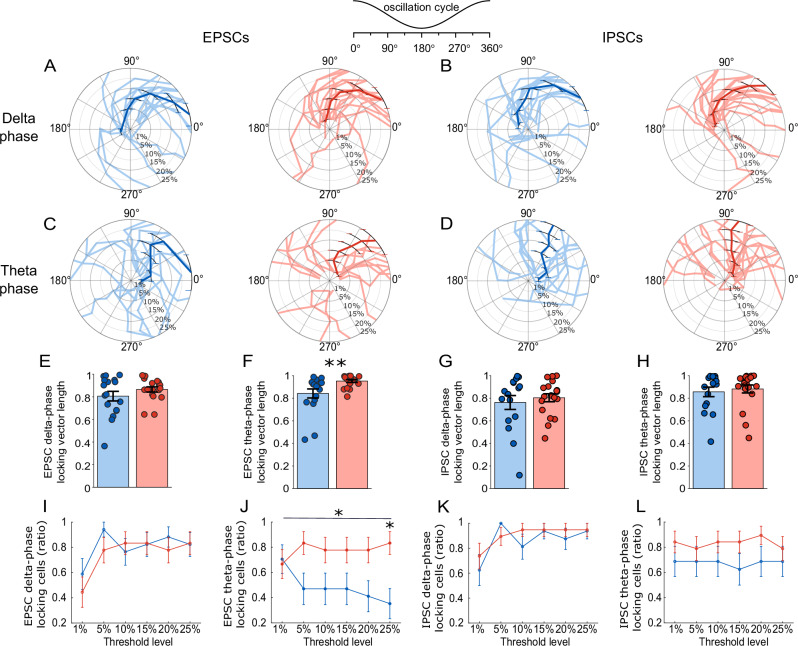
Phase-locking of excitatory and inhibitory synaptic currents to hippocampal oscillations in anesthetized young APP/PS1 mice. Polar plots showing the preferred phase of excitatory (EPSC; **A** and **C**) and inhibitory (IPSC; **B** and **D**) postsynaptic current onsets relative to delta (**A** and **B**; 1–4 Hz) and theta (**C** and **D**; 4–12 Hz) oscillations, respectively, in APP/PS1 (in red) and WT (in blue) mice. The 1%, 5%, 10%, 15%, 20%, and 25% values represent the top percentile thresholds of event-onset derivatives used to select EPSCs or IPSCs for phase-locking analysis. Each thin line in the polar plots corresponds to the circular mean phase angle for an individual cell at each threshold. Thick colored lines and symbols indicate mean phase angles with SEM. Mean phase-locking vector length (phase-locking strength) of EPSCs and IPSCs during delta (**E** and **G**) and theta (**F** and **H**) oscillations, respectively. APP/PS1 mice (in red) exhibited significantly stronger theta phase-locking of EPSCs (*P* = 0.009) than WT controls (in blue). Data are presented as mean ± SEM in bar graphs with individual values in dots. Proportion of cells in which synaptic events were significantly phase-locked to delta (**I** and **K**) or theta (**J** and **L**) across detection thresholds (top 1–25% of events). **I** and **J** for excitation (EPSCs), **K** and **L** for inhibition (IPSCs). Error bars represent SEM. Note a significantly higher proportion of cells receiving theta phase-locked EPSCs across thresholds in APP/PS1 (red) compared to WT mice (blue) in panel (**I**) (main effect of genotype, *P* = 0.0314). Post hoc pairwise *t*-tests confirmed a significant difference at the 25% threshold (*P* = 0.0168, Bonferroni-corrected). Asterisks indicate statistical significance (*P < 0.05, **P < 0.01).

We plotted the timing of these events against the phase space of the oscillation cycle (Fig. 5A–D). We compared the phase-locking strength of excitatory and inhibitory synaptic events to oscillation cycles as a measure of phase-locking magnitude. APP/PS1 mice exhibited a significantly greater mean phase-locking vector length of EPSCs to theta (Fig. 5F; 0.952 ± 0.013 for APP/PS1, *n* = 18 (12 animals); 0.842 ± 0.040 for WT, *n* = 17 (14 animals); *P* = 0.009, *F*_(1,33)_ = 7.651), but delta phase-locking strength did not differ across genotypes (Fig. 5E; 0.865 ± 0.023 for APP/PS1, *n* = 18 (12 animals); 0.806 ± 0.043 for WT, *n* = 17 (14 animals); *P* = 0.246, *F*_(1,33)_ = 1.396). In contrast, IPSCs did not show difference in their phase-locking strength, either to delta (Fig.5G; 0.804 ± 0.036 for APP/PS1, *n* = 19 (12 animals); 0.762 ± 0.062 for WT, *n* = 16 (14 animals); *P* = 0.724, *F*_(1,33)_ = 0.127), or theta (Fig.5H; 0.882 ± 0.036 for APP/PS1, *n* = 19 (12 animals); 0.856 ± 0.042 for WT, *n* = 16 (14 animals); *P* = 0.647, *F*_(1,33)_ = 0.214) oscillations.

In addition, a complementary quantitative analysis showed that APP/PS1 mice exhibited a significantly higher proportion of cells receiving theta phase-locked EPSCs than WT mice (Fig. 5J; main effect of genotype: *P* = 0.031, *F*_(1,33)_ = 5.054; genotype × threshold interaction: *P* = 0.015, *F*_(1,33)_ = 6.609; *n* = 18 (12 animals) for APP/PS1; *n* = 17 (14 animals) for WT), whereas no genotype difference was observed in cells receiving delta phase-locked EPSC (Fig. 5I; *P* = 0.550, *F*_(1,33)_ = 0.364). For inhibition, we did not find significant differences in cells harboring either delta phase-locked IPSCs (Fig.5K, P = 0.598, *F*_(1,33)_ = 0.284, *n* = 19 (12 animals) for APP/PS1; *n* = 16 (14 animals) for WT), or theta phase-locked IPSCs (Fig. 5H, *P* = 0.178, *F*_(1,33)_ = 1.895, *n* = 19 (12 animals) for APP/PS1; *n* = 16 (14 animals) for WT).

Together, these findings suggested an enhanced synchrony between excitatory synaptic events and hippocampal theta oscillations in young APP/PS1 mice, supporting the findings from prior power spectrum analysis of synaptic currents (Fig.4). Our results indicated that the observed acceleration of hippocampal oscillations in young APP/PS1 mice could be attributed to a selectively elevated synchrony of excitatory synaptic drive arriving at a higher frequency.

### Hypoactivity of hippocampal fast-spiking interneurons in awake young APP/PS1 mice

**Fig. 6:**
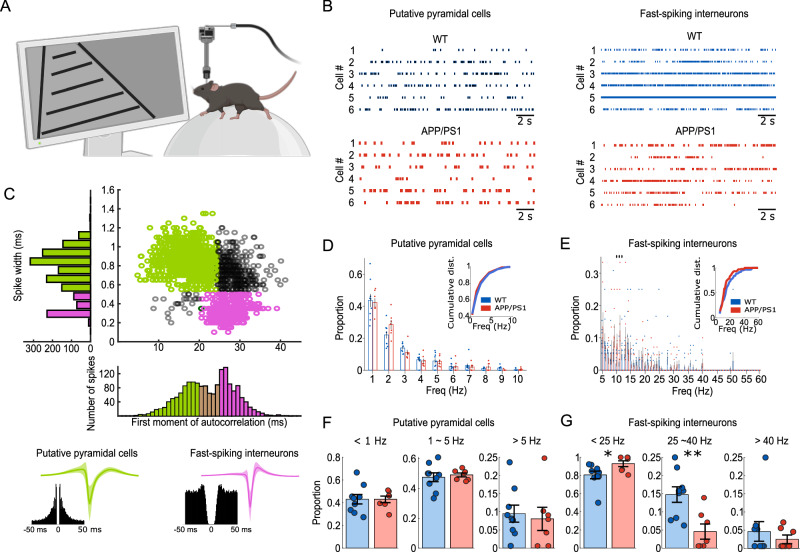
Firing properties of hippocampal neurons in awake behaving young APP/PS1 mice. (**A**) Schematic diagram showing high-throughput silicon probe recording from awake, behaving mice in a virtual reality (VR) system. Created in BioRender. Gan, J. (2026) https://BioRender.com/amnpwjg. (**B**) Example raster plots from putative pyramidal cells (left) and fast-spiking interneurons (right) from WT (top) and APP/PS1 mice (bottom), respectively. Each row of the raster represents an individual neuron, and each vertical tick represents the time of an action potential. (**C**) Classification of putative pyramidal cells (green) and fast-spiking interneurons (magenta) based on spike width and first moment of autocorrelogram properties. Below, representative autocorrelograms and average waveforms with standard deviation (shaded) are shown for each cell type. Distributions of mean firing rates across the entire recording period for putative pyramidal cells (**D**) and fast-spiking interneurons (**E**), analyzed per animal, respectively. Insets show the averaged cumulative distributions of firing frequencies for APP/PS1 (red) and WT (blue) mice. Pyramidal cells showed no significant difference between genotypes, whereas fast-spiking interneurons exhibited an altered distribution with an increased fraction of low-frequency firing population in APP/PS1 mice. Each frequency range is tested by *t*-test, and * indicates the frequency range at which the distribution is statistically different. (*P* = 0.043 at 10 Hz; *P* = 0.049 at 11 Hz; *P* = 0.033 at 12 Hz). **F**, **G** Quantification of the proportion of CA1 neurons within different firing-rate categories. Proportions were quantified per animal, shown in bar charts. **F** Putative pyramidal cells did not show a significant genotype difference across different firing rate ranges (<1 Hz, 1–5 Hz, and >5 Hz). **G** Putative fast-spiking interneurons. Note an increased proportion of low-firing (<25 Hz, *P* = 0.023) and a decreased proportion of medium-firing populations (25–40 Hz, *P* = 0.0004) in APP/PS1 mice. High-firing proportion remained unchanged. Data are presented as mean ± SEM in bar graphs with individual values in dots. Asterisks indicate statistical significance (*P < 0.05, **P < 0.01, ***<0.001).

To avoid potential confounding effects of general anesthesia on cellular physiology and network-level properties, we sought to validate our findings in drug-free, behaving animals. Head-restrained young APP/PS1 mice (3–5 months old) were trained to run on a linear treadmill within a virtual reality environment (Fig. 6A). Using high-density silicon probe recordings, we simultaneously captured single-neuron activity (Fig.6B) and LFPs in the hippocampus during task performance. In total, we recorded 757 neurons from 34 recording sessions in 7 APP/PS1 mice and 975 neurons from 40 recording sessions in 9 WT controls.

We initiated our analysis at the single-cell level to investigate whether hippocampal pyramidal cells and interneurons are differentially affected by the accumulation of Aβ species. To this end, we identified putative pyramidal cells and fast-spiking interneurons from well-clustered single units using established criteria^40–43^ (see details in Methods). This classification resulted in the identification of 948 putative pyramidal cells, 264 putative fast-spiking interneurons, and 520 unclassified units (Fig. 6C).

We first analyzed the mean firing rates of putative pyramidal cells and fast-spiking interneurons recorded throughout the entire session. The distribution of mean firing rates for putative pyramidal cells was similar between APP/PS1 and WT mice (Fig. 6D), indicating that the overall excitatory neuronal firing activity remained stable in APP/PS1 mice. In contrast, fast-spiking interneurons exhibited an altered firing rate distribution (Fig. 6E), with APP/PS1 mice showing a trend of increased proportion of interneurons firing at lower frequencies (e.g. <25 Hz), resulting in a leftward shift of the cumulative distribution. To further examine potential animal state-dependent differences, we analyzed firing rate distributions separately during running and resting periods. Similar to the full-session analysis, the firing rate distribution of putative pyramidal cells remained comparable between genotypes in both behavioral states (Supplementary Fig. 4A, C, left). In contrast, putative fast-spiking interneurons exhibited altered firing rate profiles during both running and resting, with APP/PS1 mice showing a trend of increased proportion of lower-frequency firing interneurons in both behavioral states (Supplementary Fig. 4B, D).

Based on the observed firing rate distribution patterns, we categorized the mean firing rates into three groups. The proportion of putative pyramidal cells was comparable across low (<1 Hz, 0.430 ± 0.029 for APP/PS1, *n* = 7 animals; 0.431 ± 0.041 for WT, *n* = 9 animals; *P* = 0.986, *F*_(1,14)_ = 0.0003), medium (1–5 Hz, 0.490 ± 0.013 for APP/PS1, *n* = 7 animals; 0.475 ± 0.030 for WT, *n* = 9 animals; *P* = 0.650, *F*_(1,14)_ = 0.215), and high (>5 Hz, 0.080 ± 0.032 for APP/PS1, *n* = 7 animals; 0.095 ± 0.024 for WT, *n* = 9 animals; *P* = 0.695, *F*_(1,14)_ = 0.161) firing rate ranges between WT and APP/PS1 mice (Fig. 6F). In contrast, we observed notable changes in firing rate distributions in putative fast-spiking interneurons. Specifically, the proportion of low-firing-rate population (<25 Hz) was significantly increased in APP/PS1 animals compared to WT controls (0.930 ± 0.032 for APP/PS1, *n* = 7 animals; 0.806 ± 0.042 for WT, *n* = 9 animals; *P* = 0.032, *F*_(1,14)_ = 5.644), whereas the proportion of medium-firing-rate population (25–40 Hz) was decreased (0.046 ± 0.021 for APP/PS1, *n* = 7 animals; 0.148 ± 0.021 for WT, *n* = 9 animals; *P* = 0.003, *F*_(1,14)_ = 12.730). The proportion of high-firing-rate population (>40 Hz) did not differ significantly between genotypes (0.025 ± 0.012 for APP/PS1, *n* = 7 animals; 0.046 ± 0.027 for WT, *n* = 9 animals; *P* = 0.487, *F*_(1,14)_ = 0.511) (Fig. 6G). These data indicated an overall ‘hypoactivity’ of fast-spiking interneurons in drug-free, fully awake young APP/PS1 mice. Given that fast-spiking interneurons account for about 25% of total interneuron populations in the hippocampus^44,45^, and they are highly active and perisomatic-targeting, these results could explain the previous finding of an overall reduction in basal inhibitory transmission from in vivo patch-clamp recordings (Fig. 1E, L).

### Enhanced bursting of CA1 pyramidal cells in awake young APP/PS1 mice

Hyperactivity of CA1 pyramidal cells in young APP/PS1 mice has been observed by in vivo 2-photon Ca^2+^ imaging^8^. However, the cellular origins of ‘hyperactivity’ are still not fully clear. In addition, this finding was reported under general anesthesia. To overcome these limitations, we took advantage of high temporal resolution using high-throughput silicon probe electrophysiology in awake mice.

**Fig. 7:**
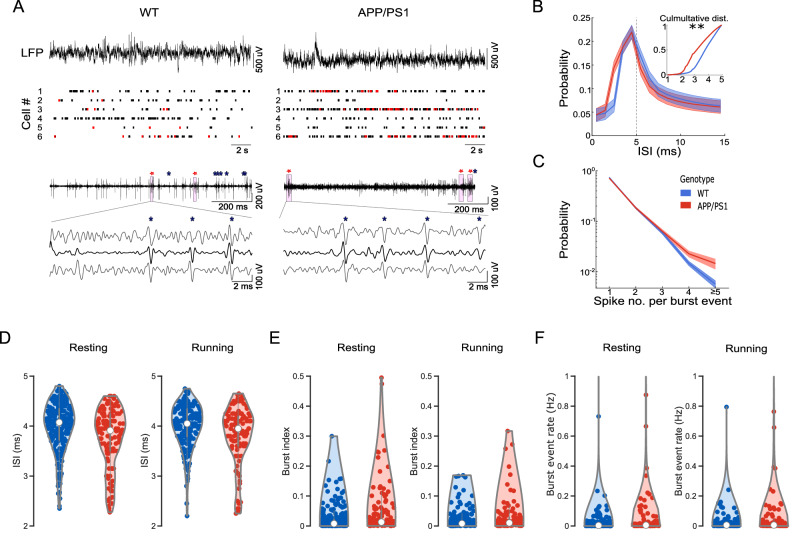
Bursting characteristics in awake behaving young APP/PS1 mice. **A** Top. Representative raw local field potential (LFP) recordings and concurrent spike raster plots from six putative CA1 pyramidal cells from WT (left) and APP/PS1 (right) mice. Note single spikes in black and bursting events in red. Bottom. Representative burst events in high-pass–filtered LFP recordings from WT (left) and APP/PS1 (right) mice. Red stars denote detected burst events; blue asterisks denote single spikes. Below, zoomed-in burst events are shown on three independent channels. **B** Interspike-interval (ISI) distribution across putative CA1 pyramidal cells at the range of 0–15 ms. APP/PS1 in red and WT in blue. Thick line represents the mean, and shading represents SEM. Inset: Cumulative ISI distribution up to 5 ms showing shorter ISIs in APP/PS1 neurons compared to WT controls (Kolmogorov–Smirnov test, *P* = 0.0026). **C** Probability of spike count per burst, demonstrating a right-shift toward multi-spike bursts in APP/PS1 neurons. Thick line represents the mean, and the shaded area represents SEM. **D** Quantification of ISIs in bursting events during resting (left) and running (right) periods, respectively. APP/PS1 in red, and WT in blue. Violin plot showing the mean, individual values, and their distribution. APP/PS1 neurons exhibit a trend of shorter ISIs in both states. **E** Burst index during resting (left) and running (right) conditions with APP/PS1 in red and WT in blue. APP/PS1 neurons exhibit a trend of higher burst index during resting. **F** Burst event rate during resting (left) and running (right) with APP/PS1 in red and WT in blue. APP/PS1 mice show a trend of higher burst event rate at rest. Asterisks indicate statistical significance (*P < 0.05, ** *P* < 0.01, *** *P* < 0.001).

We analyzed action potential firing patterns in putative pyramidal cells from our experiments (Fig. 7A). To quantify bursting characteristics, we examined the interspike interval (ISI) distribution across all classified pyramidal cells. APP/PS1 neurons exhibited a trend of shorter ISIs than WT controls, reflected predominantly in the <5 ms range (Fig. 7B, inset; Kolmogorov–Smirnov test, *P* = 0.0026). Based on this observation, we quantified bursting properties using the threshold of ISI shorter than 5 ms. The ratio of bursting cells did not significantly differ between groups (0.784 ± 0.044 for APP/PS1, *n* = 7 animals; 0.777 ± 0.029 for WT, *n* = 9 animals, *P* = 0.874, *F*_(1,14)_ = 0.026). However, the distribution of spike counts per burst event suggested that APP/PS1 neurons displayed a trend of increased intra-burst spike number compared to WT controls (Fig. 7C).

During resting periods, CA1 pyramidal neurons in APP/PS1 mice showed a trend of shorter ISI during bursting compared to WT controls (Fig. 7D, left; 3.753 ± 0.053 ms for APP/PS1, *n* = 130 (7 animals); 3.986 ± 0.033 ms for WT, *n* = 229 (8 animals)). In addition, there was a trend of increase in burst index (Fig. 7E, left; 0.046 ± 0.007 for APP/PS1, *n* = 130 (7 animals); 0.023 ± 0.002 for WT, *n* = 229 (8 animals), and in burst event rate (Fig. 7F, left; 0.090 ± 0.003 Hz for APP/PS1, *n* = 130 (7 animals); 0.022 ± 0.006 Hz for WT, *n* = 229 (8 animals)). Wilcoxon rank-sum test showed significant differences between groups for ISI (*P* = 0.0005), burst index (*P* = 0.0021), and burst event rate (*P* = 0.0183), but these effects were not significant when using a generalized linear mixed model (GLMM) that accounted for inter-session and inter-animal variability (ISI: *P* = 0.170, *F*_(1,357)_ = 1.892; burst index: *P* = 0.084, *F*_(1,357)_ = 2.995; burst event rate: *P* = 0.234, *F*_(1,357)_ = 1.421).

During running periods however, a similar trend of shorter ISI within bursts was observed in APP/PS1 mice (Fig.7D, right; 3.806 ± 0.056 ms for APP/PS1, *n* = 114 (7 animals); 3.999 ± 0.003 ms for WT, *n* = 200 (9 animals)). Again, Wilcoxon rank-sum test showed a significant effect between groups (*P* = 0.0084), but it was not the case when inter-session and inter-animal variability were considered by GLMM (*P* = 0.217, *F*_(1,312)_ = 1.534). Burst index (Fig.7E, right; 0.029 ± 0.005 for APP/PS1, n = 114 (7 animals); 0.019 ± 0.002 for WT, *n* = 200 (9 animals); *P* = 0.202, *F*_(1,312)_ = 1.631) and burst event rate (Fig. 7F, right; 0.084 ± 0.043 Hz for APP/PS1, *n* = 114 (7 animals); 0.0198 ± 0.005 Hz for WT, *n* = 200 (9 animals); *P* = 0.526, *F*_(1,312)_ = 0.403) remained comparable across genotypes.

Overall, our findings suggested that young APP/PS1 mice exhibited a trend towards higher bursting frequency and likely more intra-burst spikes when bursts were generated in hippocampal CA1 pyramidal cells than their WT counterparts. In addition, there was a trend of more frequent bursting activity in CA1 pyramidal cells in APP/PS1 mice during resting. However, these phenotypes may be subtle, and variability across animals and sessions may contribute to the observed differences. Furthermore, these phenotypes may be slightly more prominent in faster and stronger bursts, as no significant difference in bursting properties was observed if the ISI threshold was extended to 10 ms or 15 ms (Supplementary Fig. 5).

### Acceleration of hippocampal oscillations in awake behaving young APP/PS1 mice

Given our earlier findings of accelerated hippocampal oscillations in young APP/PS1 mice under general anesthesia (Fig.3), we next examined whether similar findings held in awake, drug-free conditions. To avoid the influence of the aperiodic component in the LFP, we again computed the power spectrum of the periodic component by subtracting the aperiodic component from the raw LFP spectrum^36^. The magnitude of the aperiodic component was indistinguishable across genotypes (Supplementary Fig. 2B, C, E, F). The resulting aperiodic-subtracted power spectrum was used in the following analysis.

**Fig. 8:**
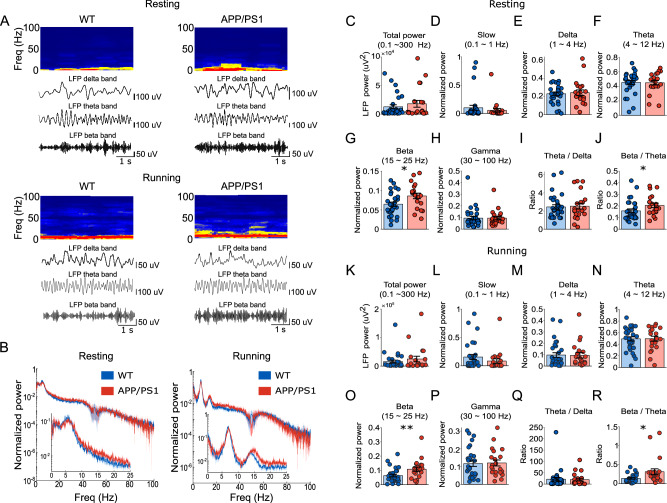
Characteristics of hippocampal oscillations in awake behaving young APP/PS1 mice. **A** Time-frequency representation of hippocampal LFP activity in WT (left) and APP/PS1 (right) mice during resting (upper panel) and running conditions (lower panel), respectively. The color scale represents power intensity, with red indicating the highest power and blue indicating the lowest power. Representative band-filtered LFP traces for delta (1–4 Hz), theta (4–12 Hz), and beta (15–25 Hz) frequencies are shown. **B** Normalized power spectral density of hippocampal LFPs recorded during resting (left) and running (right) periods in WT and APP/PS1 mice. Thick line represents the mean, and the shaded area represents SEM. Insets show zoomed-in pattern between 0 to 25 Hz. **C**–**H** Absolute total power and normalized power in different frequency bands (slow: 0.1–1 Hz, delta: 1–4 Hz, theta: 4–12 Hz, beta: 15–25 Hz, gamma: 30–100 Hz) during resting periods. APP/PS1 mice exhibited a significantly increased proportion of beta power compared to WT mice (*P* = 0.022). Ratios of theta/delta (**I**) and beta/theta (**J**) during resting periods. Note a significantly increased beta/theta ratio (*P* = 0.035). **K**–**P** Same as (**C**–**H**) but during running periods. Note an increased beta power proportion in APP/PS1 mice (*P* = 0.009). **Q**, **R** Same as (**I**–**J**) but during running periods. Note a significantly increased beta/theta ratio (*P* = 0.015). Data are presented as mean ± SEM in bar graphs with individual values in dots. Asterisks indicate statistical significance (*P < 0.05, **P < 0.01).

We first analyzed the power spectrum of hippocampal LFPs recorded when the animals briefly rested between runs on the linear track (Fig. 8A, upper and B, Left). No significant difference was observed between APP/PS1 and WT mice in total power (0.1–300 Hz) (Fig.8C; 18062.00 ± 5956.20 µV^2^ for APP/PS1, *n* = 21 (7 animals); 12916.00 ± 3443.10 µV^2^ for WT, *n* = 30 (9 animals); *P* = 0.939, *F*_(1,49)_ = 0.006). However, APP/PS1 mice exhibited a significant increase of normalized power in beta band (15–25 Hz) (Fig.8G; 0.086 ± 0.007 for APP/PS1, *n* = 21 (7 animals); 0.065 ± 0.005 for WT, *n* = 30 (9 animals); *P* = 0.022, *F*_(1,49)_ = 5.559), whereas the proportions of slow (0.1–1 Hz, Fig.8D, 0.055 ± 0.033 for APP/PS1, *n* = 21 (7 animals); 0.100 ± 0.043 for WT, *n* = 30 (9 animals); *P* = 0.567, *F*_(1,49)_ = 0.332), delta (1–4 Hz, Fig.8E; 0.241 ± 0.030 for APP/PS1, *n* = 21 (7 animals); 0.229 ± 0.019 for WT, *n* = 30 (9 animals); *P* = 0.719, *F*_(1,49)_ = 0.131), theta (4–12 Hz; Fig. 8F; 0.450 ± 0.030 for APP/PS1, *n* = 21 (7 animals); 0.454 ± 0.027 for WT, *n* = 30 (9 animals); *P* = 0.580, *F*_(1,49)_ = 0.311), and gamma (30–100 Hz; Fig.8H; 0.094 ± 0.015 for APP/PS1, *n* = 21 (7 animals); 0.094 ± 0.0150 for WT, *n* = 30 (9 animals); *P* = 0.658, *F*_(1,49)_ = 0.199) remained unchanged. Interestingly, the ratio of beta/theta showed a significant increase in APP/PS1 mice (Fig. 8J; 0.205 ± 0.019 for APP/PS1, *n* = 21 (7 animals); 0.158 ± 0.016 for WT, *n* = 30 (9 animals); *P* = 0.035, *F*_(1,49)_ = 4.721), while theta/delta (Fig. 8I; 2.537 ± 0.323 for APP/PS1, *n* = 21 (7 animals); 2.456 ± 0.244 for WT, *n* = 30 (9 animals); *P* = 0.961, *F*_(1,49)_ = 0.002) was not significantly different between groups.

We further analyzed the power spectrum of hippocampal LFPs recorded when animals actively ran in the linear track (Fig.8A, lower and B, right). Again, no significant difference was observed between APP/PS1 and WT mice in total power during running (Fig.8K; 25142 ± 9666.7 µV^2^ for APP/PS1, *n* = 21 (7 animals); 16104 ± 5085.3 µV^2^ for WT, *n* = 30 (9 animals); *P* = 0.969, *F*_(1,49)_ = 0.002). However, APP/PS1 mice still exhibited an increase of normalized power in beta band (Fig.8O; 0.108 ± 0.015 for APP/PS1, *n* = 21 (7 animals); 0.063 ± 0.009 for WT, *n* = 30 (9 animals); *P* = 0.009, *F*_(1,49)_ = 7.408), whereas the proportions of slow (Fig.8L, 0.084 ± 0.040 for APP/PS1, *n* = 21 (7 animals); 0.154 ± 0.045 for WT, *n* = 30 (9 animals); *P* = 0.576, *F*_(1,49)_ = 0.317), delta (Fig.8M; 0.094 ± 0.024 for APP/PS1, *n* = 21 (7 animals); 0.097 ± 0.020 for WT, *n* = 30 (9 animals); *P* = 0.979, *F*_(1,49)_ = 0.0007), theta (Fig.8N; 0.495 ± 0.043 for APP/PS1, *n* = 21 (7 animals); 0.492 ± 0.036 for WT, *n* = 30 (9 animals); *P* = 0.848, *F*_(1,49)_ = 0.037), and gamma (Fig.8P; 0.120 ± 0.017 for APP/PS1, *n* = 21 (7 animals); 0.117 ± 0.015 for WT, *n* = 30 (9 animals); *P* = 0.615, *F*_(1,49)_ = 0.256) remained unchanged. Interestingly, although the ratio of theta/delta (Fig.8Q; 19.690 ± 5.937 for APP/PS1, *n* = 21 (7 animals); 21.578 ± 7.594 for WT, *n* = 30 (9 animals); *P* = 0.848, *F*_(1,49)_ = 0.037) was not significantly different between groups, the ratio of beta/theta showed again a significant increase in APP/PS1 mice (Fig.8R; 0.316 ± 0.077 for APP/PS1, *n* = 21 (7 animals); 0.141 ± 0.019 for WT, *n* = 30 (9 animals); *P* = 0.015, *F*_(1,49)_ = 6.431). Further analyses examining peak-frequency ratio between beta and theta oscillations and power–power correlation (Supplementary Fig. 6) indicated the theta harmonic component in the observed beta oscillation range (15–25 Hz) was limited in these recordings.

Together, LFPs recorded during both resting and running in awake animals reinforced the observation that young APP/PS1 mice exhibited a higher proportion of faster hippocampal oscillations, though at slightly different frequency ranges compared to results from anesthesia.

## Discussion

This paper provides a quantitative analysis of the neurophysiological underpinnings of ‘early hippocampal hyperactivity’ in vivo, in a well-established amyloidopathy model of AD, the APP/PS1 mice. Our results indicate that at the single-cell level, 1) basal inhibitory synaptic input is reduced in pyramidal neurons but their intrinsic properties are largely unchanged; 2) pyramidal neurons show a trend of stronger bursts; 3) putative fast-spiking interneurons exhibit hypoactivity. At the neural network level, our data suggest: 1) a shift of hippocampal oscillation towards higher frequency ranges, albeit no alteration in total oscillatory strength; 2) selectively enhanced synchrony of synaptic excitation at higher oscillatory frequency could explain accelerated hippocampal oscillations. Whilst we have the caveat that our study was performed across anaesthetized and awake behaving APP/PS1 mice, this work demonstrates key changes in synaptic, cellular, and network hippocampal physiology in early amyloidopathy.

First, our findings directly reveal the cellular basis of ‘early hippocampal hyperactivity’ in vivo in early amyloidopathy. Hyperactivity may have simply implied that hippocampal neurons have an overall increased firing. Our results show this may not be the case. In contrast, excitatory pyramidal cells and inhibitory interneurons were differentially affected by the presence of increased Aβ species before gross plaque formation. Specifically, pyramidal cell intrinsic properties were largely unchanged, despite mildly narrowed action potentials. In awake, behaving animals, proportions of low, medium, and high firing rate pyramidal cells were largely unchanged, in agreement with a recent study using the same AD model in slightly older animals and miniature microscope Ca^2+^ imaging^46^. However, pyramidal cells did tend to fire stronger ‘bursts’, with a higher instantaneous bursting frequency and more intra-burst spikes during awake recordings. These observations could partially contribute to explaining the previously observed ‘hyperactive’ phenotype in hippocampal pyramidal neurons in young APP/PS1 mice^8^. Previous findings from Aβ plaque-bearing APP/PS1 mice also showed a profound increase in bursting in CA1 pyramidal cells in vivo^23,47^. Interestingly, however, the degree of enhanced bursting in CA1 pyramidal cells in young APP/PS1 mice appeared to be milder than that in layer 5 neocortical pyramidal cells in APP/PS1 mice of similar age^48^, and that of CA1 pyramidal cells in older, plaque-bearing APP/PS1 mice^47^. This might suggest a slight region or cell-type difference in susceptibility to disease progression. Together, our data suggest that the enhanced bursting in pyramidal cells could be a key cellular substrate of early hippocampal hyperactivity in AD.

GABAergic interneurons have been suggested to be selectively vulnerable in AD^40,48–52^. Here, our results demonstrate a ‘hypoactivity’ phenotype in hippocampal fast-spiking interneurons in awake, behaving young APP/PS1 mice. Fast-spiking interneurons, which are typically parvalbumin (PV) positive, account for about 20–30% the total interneuron population^44,45^. Given their peri-somatic axonal arborization into pyramidal cells, it is conceivable that a large proportion of inhibitory synaptic inputs recorded at the soma of pyramidal cells are of PV^+^ interneuron origin. Therefore, fast-spiking interneuron hypoactivity is consistent with the profound reduction of synaptic inhibition onto pyramidal cells observed in voltage-clamp recordings in vivo. Particularly, it explains the reduced frequency of sIPSCs seen in pyramidal cells, which suggests the involvement of a presynaptic mechanism, although the density of presynaptic interneuron populations (Supplementary Fig. 7), inhibitory synapses (Supplementary Fig. 8), and myelinated GABAergic axons (Supplementary Fig. 9) were largely unaltered. We also attempted to identify putative PV^+^ basket cells within our fast-spiking cell population, but low cell number prevented drawing a clear conclusion in their firing pattern (Supplementary Fig. 10). Interestingly, a recent study has shown a very similar ‘hypoactivity’ phenotype in fast-spiking interneurons in neocortical layer 5/6 in awake young APP/PS1 mice^48^. Together with our data, these in vivo results may suggest that the early vulnerability of fast-spiking interneurons in amyloidopathy may not only be restricted to the deep layers of the neocortex, but also in deep structures, such as the hippocampus.

Second, we describe ‘early hippocampal hyperactivity’ at the functional network level in the form of accelerated hippocampal oscillations in vivo in early amyloidopathy. Compared to single-cell level alterations in neurophysiology, early markers at the functional network level may render higher translational value. Indeed, early hippocampal hyperactivity has been observed consistently in cognitively normal individuals who have a high risk of developing AD, e.g. individuals with risk genes of familial AD^18,53^, cerebral Aβ deposition^54,55^, *APOE4* carriers^18,19,21,22^, largely by fMRI scanning. Accelerated hippocampal oscillations may be able to bridge the gap between the enhanced BOLD signals and neural network activities that form the neural basis of key cognitive functions in the hippocampus, such as encoding and consolidating spatial memory. Our finding that the total strength of hippocampal oscillations is largely intact coincides with observations that young animals with sustained Aβ species before substantial plaque formation do not show overt alterations in hippocampus-related cognitive functions^56^, and typical reduction of total neural activities only occurs at a later stage in people with clinical AD^16,57^.

A recent study using the same animal model shows neocortical EEG power spectra shifts to higher frequencies at 12 months of age in both awake and sleep conditions^58^. The pattern and extent of neocortical EEG shifts across frequency ranges with increased plaque burden in this study are very similar to our findings in the hippocampus, where overt plaque burden is not formed. This contrast might suggest that accelerated oscillations might not simply be a functional alteration restricted locally in the hippocampus but a progressing phenotype that may spread across brain regions with increasing Aβ burden. In addition, another study using awake APP/PS1 animals demonstrates a very similar increased relative power of beta oscillations and a general pattern of accelerated hippocampal oscillations across age^59^. Moreover, a recent human study targeting the hippocampus using magnetoencephalography (MEG) shows elevated faster oscillatory components in prodromal AD subjects^60^, corroborating our findings from the AD model. Taken together, accelerated hippocampal oscillations at the early stage of AD may be considered as a translationally sensitive measurement that could indicate an early yet sustained phenotype at the level of network function in vivo. This might be particularly relevant due to the growing use of MEG in humans, which provides deep penetration and adequate temporal resolution for measuring hippocampal oscillations—something not achievable with EEG.

Hippocampal slow waves and delta oscillations serve as a temporal framework for information transfer (e.g., memory) between the hippocampus and neocortex via coordinated activation of cell assemblies^61,62^. Given that impaired slow wave oscillations have been reported in people living with AD^63,64^ and in AD models of Aβ plaque pathology^65–67^, and in wildtype mice receiving neocortical perfusion of Aβ peptides^67^, the reduction of slow wave LFP components seen at the early stage of the amyloidopathy model in our study may corroborate the notion that this key neural substrate for memory transfer is functionally deteriorating early on. Typically, in the hippocampus, rapid-eye-movement (REM) sleep is dominated by theta oscillations, whereas non-REM sleep is characterized by delta oscillations^68,69^. Although general anesthesia is a distinct low-arousal state from sleep, reduced slow wave, delta, and increased theta in LFP power in our study may hypothetically link to the disruption of non-REM sleep seen from alterations of EEG patterns in sleep in young APP/PS1 mice^46,66,70^, potentially to reflect mild sleep fragmentation, a high-risk factor for clinical AD in humans^71^. However, this may also simply reflect an altered ability for network synchrony during early amyloidopathy, regardless of state. Interestingly, sharp-wave ripples (SWRs), the high-frequency oscillation events in the hippocampus that also play a central role in memory consolidation and transfer^72^, did not exhibit significant alterations in their major features across both anesthetized recordings (Supplementary Fig. 11C–F) and resting periods in the awake condition (Supplementary Fig. 11G–J). The unaffected SWR properties in young APP/PS1 animals before gross plaque deposition contrast with profound SWR deficits in a different plaque-bearing amyloidopathy model (5XFAD mice) under awake resting^40^ and during sleep in plaque-bearing APP/PS1 mice^73^, as well as a knock-in model of amyloidopathy (APP^NL-G-F^ mice)^74^. These differences might suggest that the SWR impairment in amyloidopathy could be progressive. Sustained presence of Aβ species might not be sufficient, and Aβ burden may need to accumulate to a threshold before circuit mechanisms that underlie normal SWR generation start breaking down.

Finally, by exploiting simultaneous patch-clamp and LFP recording in vivo, we unravel the synaptic mechanism underlying accelerated hippocampal oscillations in early amyloidopathy. Oscillations typically reflect synchronous firings of neuronal assemblies^75^. For relatively slower oscillatory events such as delta, theta, and beta (e.g. from 0.1 to 25 Hz), their magnitude, measured by LFP power, depends largely on the strength of synchronized synaptic excitation (current generator), whereas synaptic inhibition typically provides a pace-making function in orchestrating synchronous firings of principal cells (rhythm generator)^76,77^. Within this framework, unchanged total power of oscillation-associated synaptic excitation may explain the largely unaltered total power of LFP. Although basal-level synaptic inhibition is reduced, the overtly unchanged total power of oscillation-associated inhibition may suggest the rhythmic inhibitory drive required for pace-making is not yet deteriorated and still capable of orchestrating principal cell firing.

For specific components, the reduction of synaptic excitation power in the delta range and its enhancement in the theta range coincide with the reduction of delta and the increase of theta oscillations in young APP/PS1 mice. In addition, phase-locking of synaptic excitation is selectively enhanced to theta oscillations. Therefore, it would be conceivable that the selective enhancement of synchrony between excitatory currents and higher oscillatory frequencies could serve as a mechanism that drives accelerated LFP in early AD.

In summary, exploiting in vivo patch-clamp and high-throughput single-unit recordings, our results identify and validate neurophysiological mechanisms of early hippocampal hyperactivity in AD at both cellular and network levels in vivo. The observed acceleration of hippocampal oscillations may serve as a potential biomarker for early-stage AD and a therapeutic target for stabilizing neural networks, thereby preserving cognitive function before significant decline occurs.

## Methods

### Ethical approval

All experiments were carried out in strict accordance with UK laws and the regulations/guidelines of the University of Edinburgh Animal Welfare and Ethical Review Body (AWERB) for animal experimentation. Protocols were approved by the British Home Office project licence PP8564759. We have complied with all relevant ethical regulations for animal use.

### In vivo patch-clamp and LFP recording in anesthetized mice

#### Animals

Male or female 2- to 3-month-old APP_swe_/PS1ΔE9 mice and their littermate controls of C57BL/6J background were used. Mice were anesthetized by i.p. injection of 100 mg kg^−1^ ketamine (Ketamidor) and 10 mg kg^−1^ xylazine (Rompun), before mounting in a stereotaxic frame (David Kopf Instruments) and were supplied with 100% oxygen through a ventilation mask. Body temperature was continuously monitored by a rectal thermometer and maintained at 37 ± 0.5 °C by placing the animal on a heating pad (Kent Scientific, UK).

#### Surgery

The skull of the animal was exposed and dried. A small craniotomy (~2 mm diameter) was made on the right or left hemisphere to target the dorsal hippocampus according to stereotaxic coordinates at AP ≈ 1.8 mm and ML ≈ 1.5 mm (AP, anterioposterior from bregma; L, lateral from midline). Subsequently, within the craniotomy window, the dura mater was carefully cut and removed. The exposed cortical surface was superfused with HEPES-buffered extracellular solution (135 mM NaCl, 3.5 mM KCl,1.8 mM CaCl_2_, 1 mM MgCl_2_, and 5 mM HEPES; pH = 7.28 with NaOH).

#### In vivo patch-clamp recording

Patch pipettes were fabricated with a micropipette puller (P-1000; Sutter Instrument), using 1.0 mm/0.5 mm (outer diameter/inner diameter) borosilicate glass tubing (Hilgenberg) and had tip resistance values of 4–6 MΩ. For voltage-clamp recordings, pipettes were filled with a Cs^+^-based intracellular solution, containing 130 mM Cs-methanesulfonate, 2 mM KCl, 10 mM EGTA, 2 mM MgCl_2_, 2 mM Na_2_ATP,10 mM HEPES, 5 mM QX-314, and 3 mg ml^−1^ biocytin (pH adjusted to 7.28 with CsOH, 290–300 mOsm). For current-clamp recordings, pipettes were filled with an intracellular solution containing 130 mM K-methanesulfonate, 10 mM EGTA, 2 mM MgCl_2_, 2 mM Na_2_ATP, 10 mM HEPES, 2 mM KCl, and 3 mg ml^−1^ biocytin (pH adjusted to 7.28 with KOH, 290–300 mOsm). A reference electrode (Ag–AgCl) was placed on the skull near the craniotomy window. The cortical surface was immersed in HEPES-buffered extracellular solution. The pyramidal cell layer of the dorsal hippocampus was targeted using age-corrected stereotaxic coordinates (AP: 1.8–2.0 mm; ML: 1.5–2.0 mm; and DV: 1.1–1.4 mm; DV: dorsal-ventral from cortical surface). Patch pipettes were gently advanced from the craniotomy window perpendicular to the cortical surface with positive pressure (~300 mbar) applied to the pipette lumen to avoid tip plugging, until ~200 μm above the target area. Positive pressure was then reduced to ~15 mbar. Tight-seal cell-attached configuration was reached in the voltage-clamp mode, with pipette holding potential set at −70 mV to minimize holding current (typically 0 to –5 pA). After break-in, the whole-cell patch-clamp recording configuration was obtained, monitored by changes in current amplitudes in response to a 10-mV test pulse. Maximal care was taken to minimize the series resistance (Rs) during recording, which was 31 ± 1 MΩ in the present data set (range: 12–50 MΩ). Rs was carefully monitored throughout the recording session using 20-ms,10-mV hyperpolarizing test pulses applied at ~1 min intervals. EPSCs and IPSCs were recorded in the voltage-clamp configuration with the same cell held at either −70 mV or +10 mV, respectively, with alternating order. Membrane potential values reported were not corrected for liquid junction potentials.

#### Local field potential recording

LFP recording pipettes were fabricated from 1.0 mm / 0.5 mm (outer diameter / inner diameter) borosilicate glass tubing (Hilgenberg) and had open-tip resistance values of 1–3 MΩ. LFP pipettes were coated with Dil before carefully inserted into the same craniotomy as the patch pipettes, at a 25° oblique angle, in the AP direction, targeting the CA1 pyramidal cell layer of the dorsal hippocampus (AP: 1.8–2.0 mm, ML: 1.8–2.0 mm, DV:1.1–1.3 mm). Positive pressure (50–80 mbar) was applied to avoid pipette plugging. The location of the LFP pipette was visualized by histology of the pipette track and by visualization of Dil. To unequivocally determine pipette location, only a single LFP pipette was inserted per animal.

#### Data acquisition

In vivo patch-clamp and LFP recordings were made using an EPC 10 Quadro amplifier (HEKA, Germany) and an InstruTech LIH 8 + 8 data acquisition system (HEKA, Germany). Both signals were low-pass-filtered at 10 kHz (Bessel), sampled at 20 kHz, and stored using Patchmaster v2x91 software running on a PC under Windows 10.

#### Biocytin labeling and cell visualization

After recording, brains were fixed for >12 h in 4% paraformaldehyde, then stored in 0.1 M phosphate buffer solution (PBS) at 4 °C. To visualize labeled cells, the hemisphere containing the recorded cell was cut into 200-μm thick parasagittal sections using a vibratome (Leica VT 1000S, Germany), washed in 0.1 M PBS containing 0.3% Triton-X and 0.05% sodium azide (PBS-T-A), and then incubated with streptavidin Alexa Fluor® 488 (1:1000 in PBS-T-A; ThermoFisher, UK) at 4 °C overnight. Sections were then washed in 0.1 M PBS, rinsed in dH2O, and mounted using VectaShield (Vector Labs, UK). Sections were imaged using a confocal microscope (SP8, Leica, Germany).

#### Synaptic currents

Synaptic charge was calculated as the area under the curve (baseline normalized to 0, area under the curve calculated using *trapz* in MATLAB) from a 30-second recording sweep at either −70 mV or +10 mV for excitation or inhibition, respectively. E/I balance was calculated as excitation charge/inhibition charge. For the quantification of spontaneous excitatory postsynaptic currents (sEPSC) and spontaneous inhibitory postsynaptic currents (sIPSC), first, the dv/dt of the trace was calculated, and peaks were detected when exceeding a set threshold of (minimum of 1.8 for EPSC and 1.2 for IPSC, with threshold varying based on signal noise and activity level) standard deviations above the mean. A minimum amplitude threshold was set for each of these events, which varied based on signal noise (minimum of 7 pA for excitation and minimum 15 pA for inhibition). To select only individual PSC events, and not cumulative events, the start of the PSC was then selected if it was within a set number of standard deviations (varied based on levels of noise and activity, minimum of 1.5 for EPSC, 0.3 for IPSC) from baseline. The end current detected also had to be within a set range of the start value (minimum of 10 pA and 70 pA for EPSC and IPSC respective but varied depending on signal) to eliminate multiple cumulative events and detected events had to be a minimum distance apart (200 and 100 samples for EPSC and IPSC respectively but depending on signal noise and activity). From this, detected events were extracted and summarized. Average waveforms were extracted and used to calculate the mean amplitude, the rate of rise, and decay tau (fitted on 20–80% of the curve). Frequency was taken as the number of individual events, normalized per second in a 30-second sweep. The interevent interval between events was also calculated.

#### Passive and active properties

Resting membrane potential (Vm) was taken as the membrane potential immediately after break-in. Passive and active properties, including excitability measures, were calculated from the current step injections. From the −100 pA hyperpolarizing sweep, input resistance was calculated, based on the difference from Vm just prior to current injection compared to the average at steady state at the end of the current injection. A dv/dt and amplitude threshold was set to detect action potentials. From detected action potentials, the maximum dv/dt rate, action potential amplitude, peak value, threshold, width at half height, and width at threshold were calculated. Input-frequency plots were made based on the number of quantified action potentials per current injection sweep.

#### LFP signal processing

Data analysis was performed using MATLAB (R2024b, MathWorks). Mains power interference (50, 100, 150, and 250 Hz) was removed by a second-order band-stop filter. Signals were down-sampled to 2 kHz using the *resample* function, which applies an FIR anti-aliasing low-pass filter, and then band-pass filtered between 0.1 and 300 Hz using a 4th-order Butterworth filter. The raw power spectrum was obtained by *pwelch* function in MATLAB (window: 10 s, 0.1 Hz step, overlap: 50%). We parameterized the LFP power spectra (1–100 Hz) using FOOOF^36^, removed line frequencies and harmonics, fit the aperiodic component, and quantified periodic band power from the residual (“peak-only”) spectra. Absolute power was calculated as the area under the curve within each frequency band. Normalized power for slow waves (0.1–1 Hz), delta (1–4 Hz), theta (4–12 Hz), beta (15–25 Hz), and gamma (30–100 Hz) oscillations was calculated by dividing the absolute power of each frequency range by the summed power between 0.1 and 100 Hz, respectively.

#### Signal processing of synaptic currents

Data analysis was performed using MATLAB (R2024b, MathWorks). Mains power interference (50, 100, 150, and 250 Hz) was removed from synaptic current (EPSC and IPSC). Signals were down-sampled to 2 kHz, and then band-pass filtered between 0.1 and 300 Hz. Slow drifts in synaptic currents were corrected by subtracting the mean of each sweep. Power spectra were computed using the *pwelch* function (window: 2 s, 0.5 Hz step, 90% overlap). Residual line noise in the power spectral density was corrected by replacing contaminated frequency bins with the mean of neighboring bins. Absolute power was calculated as the area under the curve within each frequency band, and normalized power was obtained by dividing absolute power by the total power (0.1–300 Hz).

#### Derivative-based detection of synaptic events and phase analysis

To quantitatively examine the temporal structure and relationship between synaptic currents and hippocampal oscillations in vivo, we used a first derivative-based detection method^37,38^. Synaptic currents were smoothed using a moving average filter with a 2.5 ms time constant. Next, we calculated the first derivatives of the smoothed traces. EPSC onsets were detected as derivative minima, and IPSC onsets as derivative maxima. To account for variability in synaptic current kinetics, we analyzed subsets of data corresponding to the largest derivative peaks (top 1–25% percentiles).

For phase analysis, wideband LFP signals were filtered into delta (1–4 Hz) and theta (4–12 Hz) ranges. The Hilbert transform was applied to the band-pass–filtered signals, and each EPSC or IPSC onset was assigned a corresponding instantaneous phase value. Delta (1–4 Hz) and theta (4–12 Hz) epochs were then identified using the fBOSC method^39,78^, and a minimum duration of ≥2 cycles. Phase locking of synaptic events was assessed only during fBOSC-detected delta or theta epochs.

The circular mean phase of synaptic events was computed for each cell, and the resulting values were averaged across cells. Vector length, as a measure of phase-locking consistency, was calculated for different subsets of data representing the largest derivative peaks (top 1–25% percentiles) and then averaged across these subsets. The Rayleigh test was used to assess the non-uniformity of phase distributions of synaptic events (EPSCs and IPSCs) in each cell. The proportion of cells exhibiting significant phase locking synaptic events (*p* < 0.05) was quantified.

### High-throughput silicon probe recordings in awake, behaving animals

#### Surgical Preparation

Male or female 3- to 5-month-old APP_swe_/PS1ΔE9 mice and their littermate controls of C57BL/6J background were used. Mice were anesthetized with isoflurane (3% induction, 1% maintenance, 1 L/min in pure oxygen) and secured in a stereotaxic frame. Two steel screws (1 mm in diameter) were placed in small boreholes drilled above the cerebellum and olfactory bulb. A custom-made head-ring for subsequent head-restraint training and recording was attached to the skull, with the screws firmly fixed using bone cement (Refobacin, Biomet). Before surgery, mice received a subcutaneous injection of carprofen (120–130 µL, 20 mg/kg) and a subcutaneous supplement of 0.9% NaCl (200–300 µL). Mice were given at least seven days to recover before further procedures. After recovery, mice were handled and habituated to head-restraint on an air-cushioned styrofoam ball in a virtual reality (VR) system (JetBall, Phenosys). Following habituation, mice were trained to run in a VR linear treadmill task for liquid rewards. Once the mice demonstrated consistent running behavior after 3–4 days, they were transiently anesthetized, and two small craniotomies were drilled above the hippocampus in each hemisphere (AP: ≈−1.9 mm; ML: ≈±1.5 mm). The dura was carefully removed, and the craniotomies were sealed with silicone (Kwik-Cast, World Precision Instruments). Mice were allowed to recover for at least eight hours before electrophysiological recordings began.

#### VR linear treadmill behavior

Head-fixed mice were positioned on an air-cushioned styrofoam ball within a 270° TFT surround monitor system (JetBall, PhenoSys). Mice were trained to run through a custom-designed virtual linear corridor, covering a 150 cm running path per trial. Upon reaching the end of the corridor, they received a 50 µL liquid reward (5% sucrose water) via a spout. After a 10-second inter-trial interval, mice were teleported back to the start of the corridor to begin the next trial. Each training session lasted 15–30 min daily for at least three days. Trial events were recorded as transistor-transistor logic (TTL) pulses by the acquisition device, while locomotion data were collected using an XY-motion sensor at 50 Hz.

#### Silicon probe recordings

Mice were secured in the stereotaxic frame within the VR system and allowed to perform the linear treadmill task. Silicon probe (A4X32-Poly2–5mm-23s-200-177, NeuroNexus), containing 128 recording channels across four parallel shanks, was slowly inserted into the hippocampus through the previously prepared craniotomies. Neural signals were recorded using an Intan 128-channel head-stage (C3316, Intan Technologies) connected to an Intan RHD recording controller (C3004, Intan Technologies). Signals were sampled at 20 kHz and digitized as 16-bit signed integers.

#### Spike sorting and classification of cell types

Spike sorting was performed using Kilosort 3^79^, followed by manual curation in Phy 2^80^. Spike clusters were assigned to the channel with the largest voltage trough-to-peak amplitude, determined from the average spike waveform. Only units displaying a clear refractory period (±1.5 ms) were included in the further analysis. To classify single-unit spike clusters into putative excitatory and inhibitory neurons, single units were distinguished based on spike waveform shape and the first moment of the autocorrelogram as described previously^40–43^. First, spike width was measured by calculating the time between trough and peak from the average spike waveform of each cluster. Second, the first moment of the autocorrelogram was calculated as the center of mass along the time axis of an autocorrelogram computed with lags from 0 to 50 ms. Single units were excluded if their autocorrelograms had a peak spike count of less than 10. Putative pyramidal cells were defined as units with a spike width greater than 0.5 ms and a first moment of the autocorrelogram less than 25 ms. Putative interneurons were defined as units with a spike width less than 0.5 ms and a first moment of the autocorrelogram greater than 20 ms.

#### Identification of burst firing

Complex bursts were classified as a series of three or more spikes with inter-spike intervals of less than 5 ms. Burst event rate was calculated as the number of burst events divided by the total recording time. The burst index was defined as the ratio of bursting spikes to all spikes^81^.

#### LFP analysis

LFP signals were obtained by downsampling raw traces to 2 kHz and bandpass filtering between 0.1 and 300 Hz. LFP signals from each site were bandpass filtered (150–250 Hz), and power was calculated using the Hilbert transform. The channel with the largest mean power of filtered LFP was determined for each shank in each session and designated as the pyramidal cell layer^82,83^. The power spectral density of neural activity in the pyramidal layer was computed using the MATLAB function *pwelch* (window: 10 s; overlap: 50%). We parameterized the LFP power spectra (1–100 Hz) using FOOOF^36^, removed line frequencies and harmonics, fit the aperiodic component, and quantified periodic band power from the residual (“peak-only”) spectra. Power was calculated as the area under the curve (AUC) of the power spectral density within each frequency band: slow wave (0.1–1 Hz), delta (1– 4 Hz), theta (4–12 Hz), beta (15–25 Hz), and gamma (30–100 Hz). LFP signals were additionally analyzed during running and resting periods. Treadmill speed (sampled at 50 Hz) was aligned to the LFP using sample-and-hold interpolation. Running epochs were defined as periods with speed >5 cm·s⁻¹, whereas resting epochs required speed <1 cm·s⁻¹. Contiguous segments shorter than 2 s were discarded, and periods that did not meet either threshold were classified as ambiguous and excluded from state-resolved analyses.

### Sharp wave ripple detection

To detect sharp wave ripples (SWR), the LFP from the CA1 pyramidal cell layer was band-pass filtered in ripple ranges (100–250 Hz; 4th-order zero-phase Butterworth). The analytic amplitude (Hilbert envelope) was squared to obtain ripple-band power and smoothed with a Gaussian kernel (σ = 4 ms). Candidate SWRs were defined as contiguous periods where the smoothed envelope exceeded mean +3 SD; event bounds were then extended to the nearest crossings of the mean on both sides. Candidates were retained if their duration was 15–150 ms and if spectral specificity was high, enforced by a ripple/high-frequency power ratio ≥4 computed against a control band (250–400 Hz) filtered and enveloped identically. For each accepted event, we calculated duration, the number of ripple cycles from peaks of the ripple-filtered trace, and SWR power (Z score).

### Identification of putative PV^+^ basket cells

To identify putative PV^+^ basket cells, theta epochs were first detected using procedures similar to the previously published method^84^. Briefly, CA1 LFP (2 kHz) was segmented using a combination of the LFP theta/delta (T/D) ratio and treadmill speed. The LFP was analyzed in sliding windows (1.6-s length, 0.8-s step) using multi-taper spectral estimates (pmtm, NW = 3). Theta (4–12 Hz) and delta (2–4 Hz) band power were computed for each window, and the resulting T/D ratio was linearly interpolated to match the LFP sample times. Theta epochs were defined as periods with T/D > 2 and speed > 5 cm·s⁻¹, whereas non-theta epochs required T/D < 2 and speed < 1 cm·s⁻¹. Contiguous segments shorter than 2 s were discarded. Periods that did not satisfy either set of criteria were classified as ambiguous and excluded.

Theta and SWR modulation of interneuron activity was analyzed following the previous studies^85,86^. To assess theta phase modulation, spikes from each single unit were assigned to 18° phase bins between theta troughs (0–360°), derived using a finite impulse response (FIR) filter and the Hilbert transform. The Rayleigh test was applied to determine whether each neuron was significantly phase-locked to theta oscillations and to calculate its preferred firing phase. For SWR modulation, spike activity during SWR events was compared with activity during ‘non-theta and non-SWR’ baseline periods. To quantify peri-event firing patterns, each SWR event was divided into eight bins, centered on the SWR peak (defined as the midpoint). Baseline activity was estimated from an equivalent number of randomly selected ‘non-theta and non-SWR’ periods matched in duration. Putative PV^+^ basket cells were defined as those showing a single-peaked firing pattern surrounding the SWR peak and a theta phase-locking preference between 203° and 339°. Out of 259 putative fast-spiking interneurons, 40 (15.4%) met the criteria and were classified as putative PV^+^ basket cells.

### Immunofluorescence staining of interneurons, synapses, myelination, and Aβ plaques

Following transcardial perfusion, brains were fixed in 10% formalin for 2 h and subsequently washed 3 times in 1× phosphate-buffered saline (PBS). Brains were then dehydrated in 20% sucrose solution for 48 h, snap-frozen in isopentane, and stored in at −80 °C. Brains were sectioned into 40 µm-thick slices with a cryostat machine (CM1950, Leica Microsystems), which were kept at −20 °C in anti-freezing media (500 mL 1× PBS, 85.6 g sucrose, 1.42 g magnesium chloride hexahydrate, filled to 1 L with glycerol) until immunostaining.

Sagittal mouse hippocampal brain sections from either the left or right hemisphere, spaced 480 μm apart, were used for each staining condition. For each brain, 3 technical replicates were used. The following primary antibodies were used: parvalbumin (rabbit, PV27a, 1:1000, Swant), somatostatin (rabbit, T-4103, 1:1000, BMA Biomedicals), VGAT (guinea pig, 131004, 1:250, Synaptic Systems), gephyrin (mouse, 147021, 1:100, Synaptic Systems), MBP (rat, MCA4095, 1:300, BioRad), β-amyloid 6E10 (mouse, 803004, 1:1000, BioLegend). The secondary antibodies were as follows: Alexa Fluor 594 goat anti-mouse (A11005, 1:1000, Thermo Fisher Scientific), Alexa Fluor 488 goat anti-guinea pig (A11073, 1:1000, Thermo Fisher Scientific), Alexa Fluor 594 goat anti-rat (A11007, 1:1000, Thermo Fisher Scientific), Alexa Fluor 488 goat anti-mouse (A21121, 1:1000, Thermo Fisher Scientific), Alexa Fluor 488 goat anti-rabbit (A11008, 1:500, Thermo Fisher Scientific). The immunostaining protocols used in this study are summarized below.

### Inhibitory interneuron staining

Sections were washed 3 times in 1XPBS and blocked for 1 h (10% normal goat serum (NGS), 0.3% Triton X-100, 0.05% sodium azide in PBS). Sections were incubated with primary antibodies in a blocking solution (5% normal goat serum, 0.3% Triton X-100, 0.05% sodium azide in PBS) for two days at 4 °C. Next, sections were washed 4 times in 1XPBS and incubated with the secondary antibodies in blocking solution (3% normal goat serum, 0.3% Triton X-100, 0.05% sodium azide in PBS) overnight at 4 °C. The following day, sections were washed 3 times in 1XPBS, then 2 times in 0.1 M phosphate buffer (PB). Nuclei were stained with 4′,6-diamidino-2-phenylindole (DAPI) in PB for 1 h at room temperature.

### Myelin basic protein (MBP) and PV immunostaining

Sections were washed 2 times with 1× PBS, then blocked in blocking solution (10% NGS in PBS-Triton X-100 0.2%) for 2 h. Primary antibodies were diluted in blocking solution, and sections were incubated overnight at 4 °C. The next day, sections were washed 3 times in PBS-Triton X-100 0.2% and incubated with the secondary antibodies in blocking solution for 2 h at room temperature. Lastly, sections were washed once in PBS-Triton X-100 0.2%, counterstained with DAPI, and washed again 3 times with 1× PBS.

### Synaptic staining

Sections were washed in 1× PBS, followed by antigen retrieval with a citrate-based antigen unmasking solution (H-3300-250, 1:100, Vector Laboratories) at 95 °C for 20 min. Sections were first washed once in 1× PBS, then once in PBS-Triton X-100 0.3% (PBT), and were incubated in Image-iT® FX Signal Enhancer (I36933, Thermo Fisher Scientific) for 30 min. Sections were washed once in 1× PBS and blocked with blocking solution (10% heat-inactivated horse serum, 0.3% Triton X-100, 1× PBS) for 2 h. Primary antibodies were diluted in blocking solution, and sections were incubated for two days at 4 °C. Next, sections were washed 4 times in PBT (1 h/wash) and subsequently incubated with secondary antibodies in blocking solution overnight at 4 °C. The following day, sections were washed 3 times in PBT, counterstained with DAPI, and washed again once in PBT.

### Aβ plaque staining

Sections were washed twice in PBS, then blocked for 2 h with blocking solution (10% normal goat serum, 0.2% Triton X-100, 1× PBS). Primary antibodies were diluted in blocking solution, and brain sections were incubated overnight at 4 °C. The following day, sections were washed 3 times in PBST (0.2% Triton X-100, 1× PBS), then incubated with the secondary antibody diluted in blocking solution for 2 h. Finally, sections were washed once in PBST, then counterstained with DAPI, and washed once with PBS.

All sections were mounted onto microscopic slides using ProLong Gold Antifade Mountant (P36930, Thermo Fisher Scientific).

### Image acquisition and analysis

All images were acquired with a confocal microscope (Carl Zeiss LSM 800 with AiryScan 2, Germany), using 10×, 20×, or 63× objectives. Image processing and analysis were done with the ZEN Microscopy Software, ImageJ and Fiji software, and MATLAB.

Quantitative data are expressed as mean ± standard error of the mean (SEM). Normality of data was assessed by Shapiro–Wilk test, and parametric or non-parametric analysis was selected accordingly. The difference between groups was analyzed using two-way ANOVA to account for sex and age differences, or an unpaired *t*-test. The significance level for analysis was set to *P* < 0.05. All statistical analysis was performed using the software GraphPad Prism (GraphPad Software, USA).

### Quantification of parvalbumin and somatostatin-expressing interneurons

The hippocampal CA1 was traced using the 20×/0.45 objective and Z-stacks spaced 0.85 µm apart. Positively stained interneurons in the CA1 were quantified. Image analysis was performed using ZEN Microscopy Software. The total number of PV^+^ and SST^+^ neurons was determined manually, with neurons only being included in the quantification if they had a clearly stained soma and dendrites. Final cell density was determined by dividing the number of quantified positively stained cells by the tissue volume (area of the hippocampus multiplied by section thickness).

### Measurement of MBP and PV colocalization

Images of the CA1 were captured using a 20× objective and Z-stacks (0.85 μm interval between images) and reconstructed with maximum intensity projection. Tile scanning was used to acquire images covering the CA1 area of the hippocampus. Analysis was performed using ImageJ and Fiji as previously described. Automated thresholding using Otsu’s method was performed on the output images. Immunoreactivity for MBP and PV was determined as the number of signal-positive pixels in the thresholded images. Overlap between the positive MBP and PV signals was determined using the image calculator function. For MBP colocalisation to PV-expressing interneurons, the number of MBP^+^ and PV^+^ pixels was quantified and expressed as a percentage of total PV signal-positive pixels.

### Quantification of synapses

Images covering the pyramidal layer were captured using a 63× objective and Z-stacks (acquired at 0.2 μm intervals). Representative images of the CA1 region were captured with a 63× objective. Acquired images were divided into 10 μm × 10 μm regions of interest, followed by cropping and segmentation using automated thresholding in ImageJ. Pre- and post-synaptic objects were quantified with MATLAB using a custom script. Obtained values were expressed as synapses/mm^3^ per CA1 area (pyramidal and dendritic) and averaged for each brain.

### Quantification of Aβ plaques

Images covering the whole brain section were captured using a 10× objective and Z-stacks (acquired at 1.50 μm intervals). Positively stained Aβ plaques in the hippocampus and neocortex were quantified. Image analysis was performed using ZEN Microscopy Software. The total number of Aβ plaques was determined manually. Final plaque density was determined by dividing the number of quantified Aβ plaques by the tissue volume (area of the hippocampus multiplied by section thickness).

### Statistics and reproducibility

Data are presented as means ± standard error of the mean (SEM). Group comparisons between APP/PS1 and WT genotypes were performed using a rank-based (non-parametric equivalent) generalized linear mixed model (GLMM), where genotype is treated as a fixed effect, animal and session, if appropriate, as random effects. For analyses involving repeated measurements across multiple within-cell thresholds (e.g., proportions of phase-locked cells across derivative-peak percentiles), a repeated-measures ANOVA was performed using MATLAB’s *fitrm* and *ranova* functions, with genotype as a between-subject factor and threshold as a within-subject factor. The Wilcoxon rank-sum test or t-test, when appropriate, was also used to assess differences between groups when specified. Circular uniformity in phase relationship analysis was examined using the Rayleigh test. The Kolmogorov–Smirnov test was used for cumulative distributions. All statistical analyses were conducted using MATLAB software. Statistical significance was set at *P* < 0.05. Statistical significance is indicated throughout the paper as follows: *(*P* < 0.05), **(*P* < 0.01), and ***(*P* < 0.001). Statistical details are provided in the supplementary data file.

### Reporting summary

Further information on research design is available in the Nature Portfolio Reporting Summary linked to this article.

## Supplementary information

Supplementary Information

Description of Additional Supplementary Files

Supplementary Data

Reporting Summary

## Data Availability

Numerical source data for graphs and quantitative comparisons presented in this paper are deposited at Mendeley Data: 10.17632/dpy4jt7d7v.1^87^. Any additional information required to reanalyze the data will be available from the corresponding author on reasonable request.

## Code availability

MATLAB code to reproduce graphs and quantitative comparisons presented in the paper is deposited at Mendeley Data: 10.17632/9srsh85r7p.1^88^. Any additional information required to reanalyze the data will be available from the corresponding author on reasonable request.
