# Supplementary material for: Reduced inhibition, bursting, and accelerated oscillations drive early hippocampal hyperactivity in Alzheimer’s disease in vivo
Source: Commun Biol. 2026 Mar 28;9:688. doi: 10.1038/s42003-026-09918-y (PMC13195132; doi:10.1038/s42003-026-09918-y)
Supplement: Supplementary file 1 — Supplementary Information [file 42003_2026_9918_MOESM1_ESM.pdf]

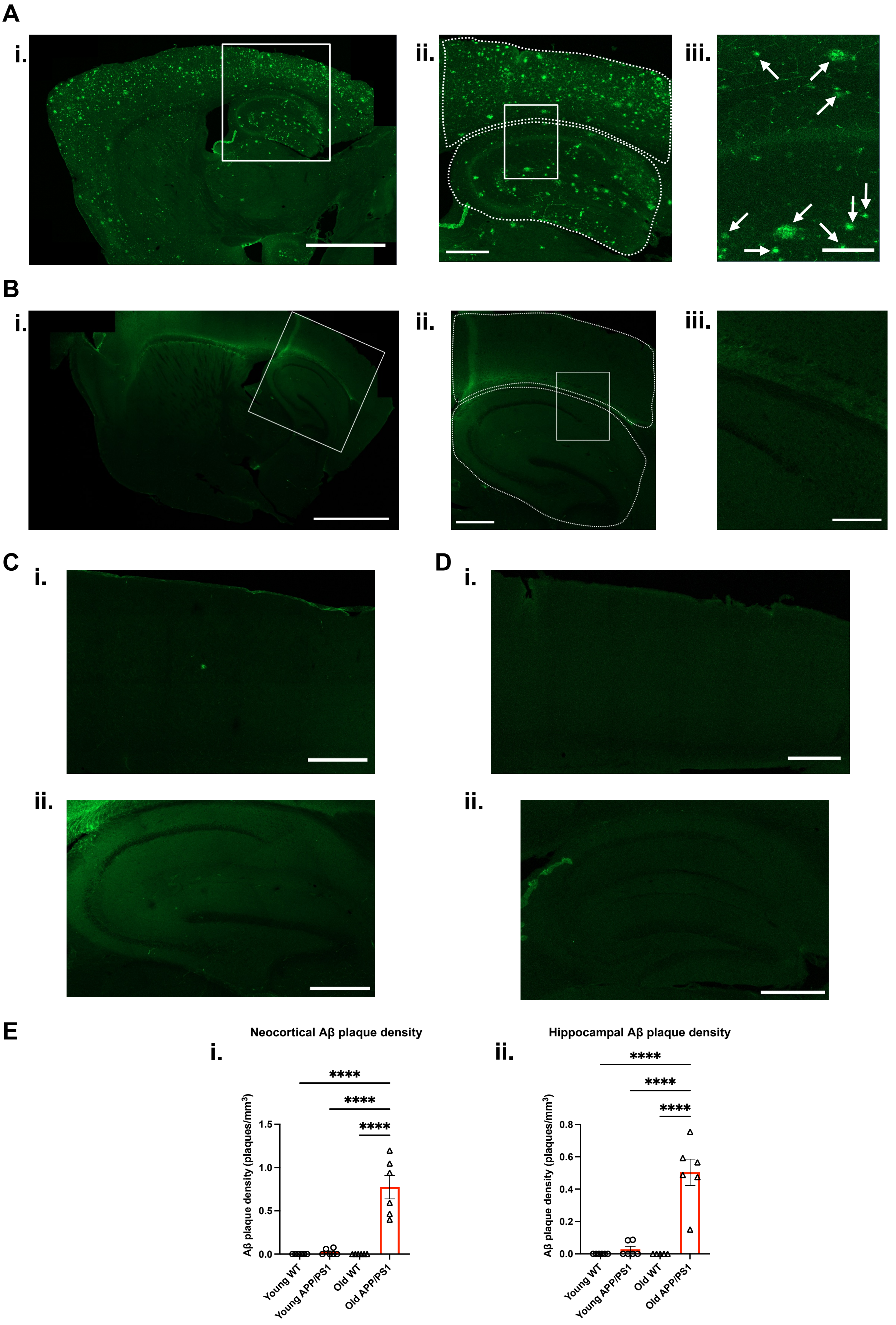

Supplementary Figure 1 Aβ plaque accumulation in the hippocampus and neocortex in young (3–5.5 months) and old (11–13 months) APP/ PS1 mice

(A) i. Representative image of Aβ plaque accumulation in old APP/PS1 mice. Scale bar: 2000 μm. **ii.** Inset image of framed area in (**i.**) showing an example of the surface areas used for quantification surrounded by a dotted line (top: neocortex, bottom: hippocampus). Scale bar: 500 μm. **iii.** Inset image of framed area in (**ii.**) with arrows indicating Aβ plaques. Scale bar: 200 μm. The β-amyloid 6E10 antibody was used to detect Aβ plaque.

(B) Same as in (**A**), but from age-matched (old) wild-type controls.

(C) Representative image of the absence of gross Aβ plaque accumulation in young APP/PS1 mice in the (**i.**) neocortex, and (**ii.**) hippocampus. Scale bar: 500 μm.

(D) Same as in (**C**), but from age-matched (young) wild-type controls.

(E) Data showing Aβ plaque density in the **i.** neocortex and **ii.** hippocampus. Density was expressed as the number of plaques per mm^3. Statistical analysis was done with a two-way ANOVA. Error bars represent mean ± SEM, **** $P < 0.0001$.

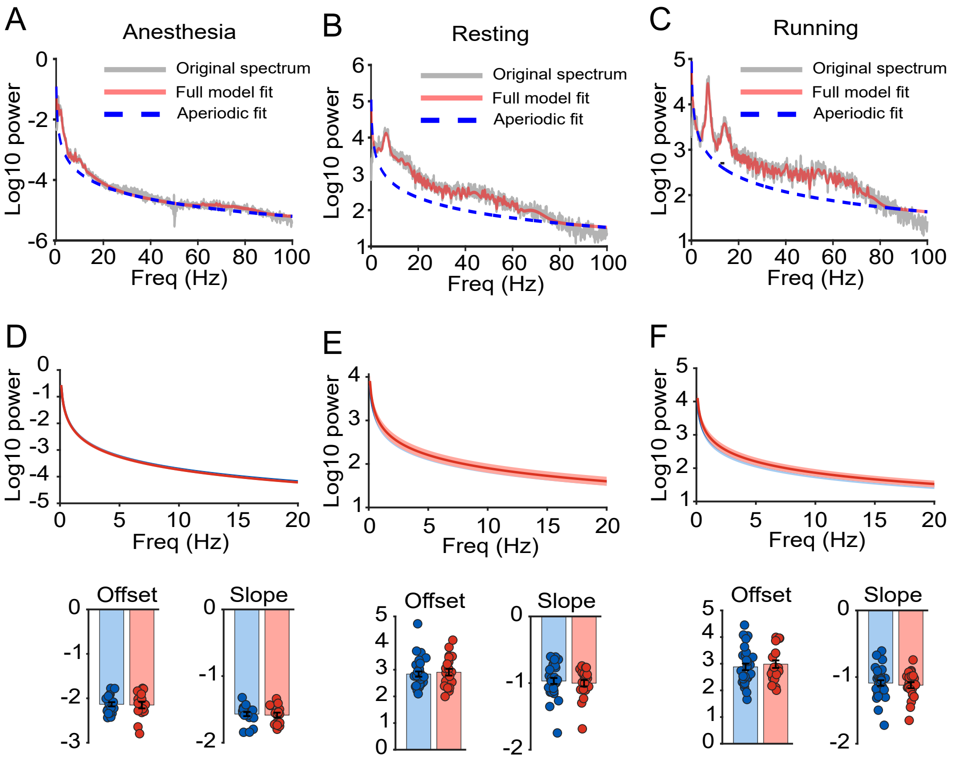

Supplementary Figure 2 Aperiodic (1/f) components of LFP power spectra in young WT and APP/PS1 mice across behavioral states

(A–C) FOOOF power spectrum models for anesthesia (**A**), resting (**B**), and running (**C**) conditions. Grey traces show the original log power spectra, red lines the full FOOOF model fits, and blue dashed lines the aperiodic (1/f) component.

(**D**–**F**) Aperiodic (1/f) fits of the power spectra (top; 1–20 Hz) and extracted aperiodic parameters (bottom) for WT (blue) and APP/PS1 (red) mice during patch (**D**), resting (**E**), and running (**F**) conditions. Shaded curves represent mean ± SEM across recordings.

For the anesthesia condition (**D**), offset values did not differ between groups (WT = −2.135 ± 0.048, n = 19 animals; APP/PS1 = −2.151 ± 0.076, n = 15 animals; $P = 0.826$, $F_{(1,32)} = 0.049$). Exponent values were also comparable (WT = −1.570 ± 0.031; APP/PS1 = −1.586 ± 0.037; $P = 0.773$, $F_{(1,32)} = 0.085$).

For the resting condition (**E**), offset values were similar between groups (WT = 2.882 ± 0.125, n = 30 recordings from 9 animals; APP/PS1 = 2.984 ± 0.142, n = 21 recordings from 7 animals; $P = 0.665$, $F_{(1,49)} = 0.189$), and no group differences were observed for the exponent (WT = −1.090 ± 0.042; APP/PS1 = −1.122 ± 0.045; $P = 0.657$, $F_{(1,49)} = 0.200$).

For the running condition (**F**), offset values remained comparable (WT = 2.837 ± 0.099, n = 30 recordings from 9 animals; APP/PS1 = 2.906 ± 0.124, n = 21 recordings from 7 animals; $P = 0.781$, $F_{(1,49)} = 0.078$), and exponent values were also similar (WT = −0.969 ± 0.045; APP/PS1 = −1.002 ± 0.048; $P = 0.614$, $F_{(1,49)} = 0.257$). Data are presented as mean ± SEM.

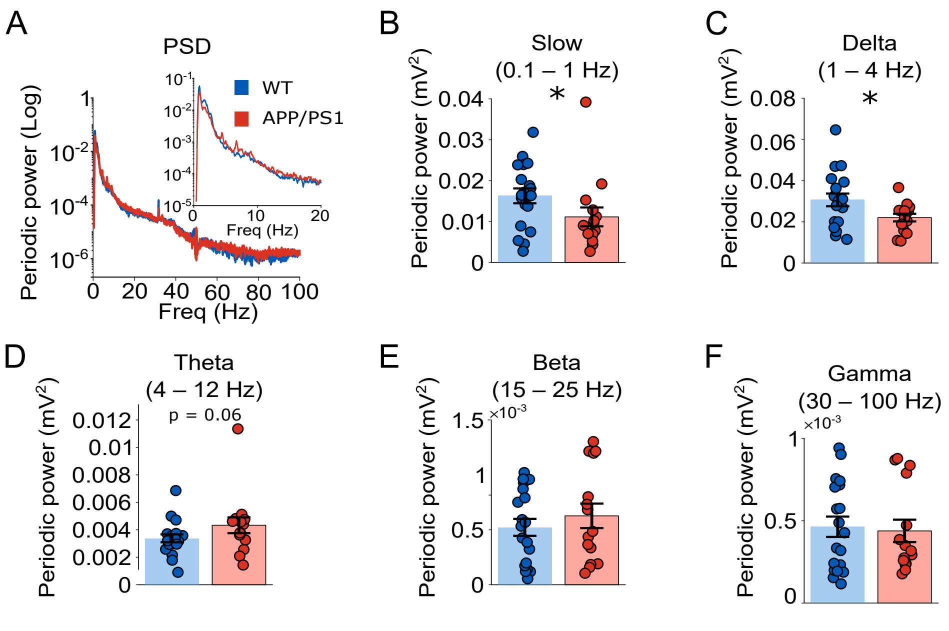

Supplementary Figure 3 Comparison of LFP periodic power between young WT and APP/PS1 mice under anesthesia

(A) Power spectral density (PSD) for WT and APP/PS1 mice.

(B) Slow-band (0.1–1 Hz) periodic power for WT and APP/PS1 mice. GLMM modeling showed a significant group effect (WT = 0.016 ± 0.002 mV^2, APP/PS1 = 0.011 ± 0.002 mV^2; $P = 0.028$, $F_{(1,32)} = 5.31$).

(C) Delta-band (1–4 Hz) periodic power. APP/PS1 mice showed significantly reduced delta power (WT = 0.031 ± 0.003 mV^2, APP/PS1 = 0.022 ± 0.002 mV^2; $P = 0.031$, $F_{(1,32)} = 5.12$).

(D) Theta-band (4–12 Hz) periodic power. A trend toward increased theta power was observed in APP/PS1 mice (WT = 0.0034 ± 0.0003 mV^2, APP/PS1 = 0.0043 ± 0.0006 mV^2; $P = 0.0624$, $F_{(1,32)} = 3.73$).

(E) Beta-band (15–25 Hz) periodic power showed no significant differences between groups (WT = $5.22\times10^{-4} \pm 7.72\times10^{-5}$ mV^2, APP/PS1 = $6.27\times10^{-4} \pm 1.11\times10^{-4}$ mV^2; $P = 0.529$, $F_{(1,32)} = 0.404$).

(F) Gamma-band (30–100 Hz) periodic power also showed no group effect (WT = $4.63\times10^{-4} \pm 6.18\times10^{-5}$ mV^2, APP/PS1 = $4.38\times10^{-4} \pm 6.80\times10^{-5}$ mV^2; $P = 0.987$, $F_{(1,32)} = 2.9\times10^{-4}$).

Data are presented as mean ± SEM (n = 19 WT, 15 APP/PS1). Asterisks indicate statistical significance (* $P < 0.05$, ** $P < 0.01$, *** $P < 0.001$).

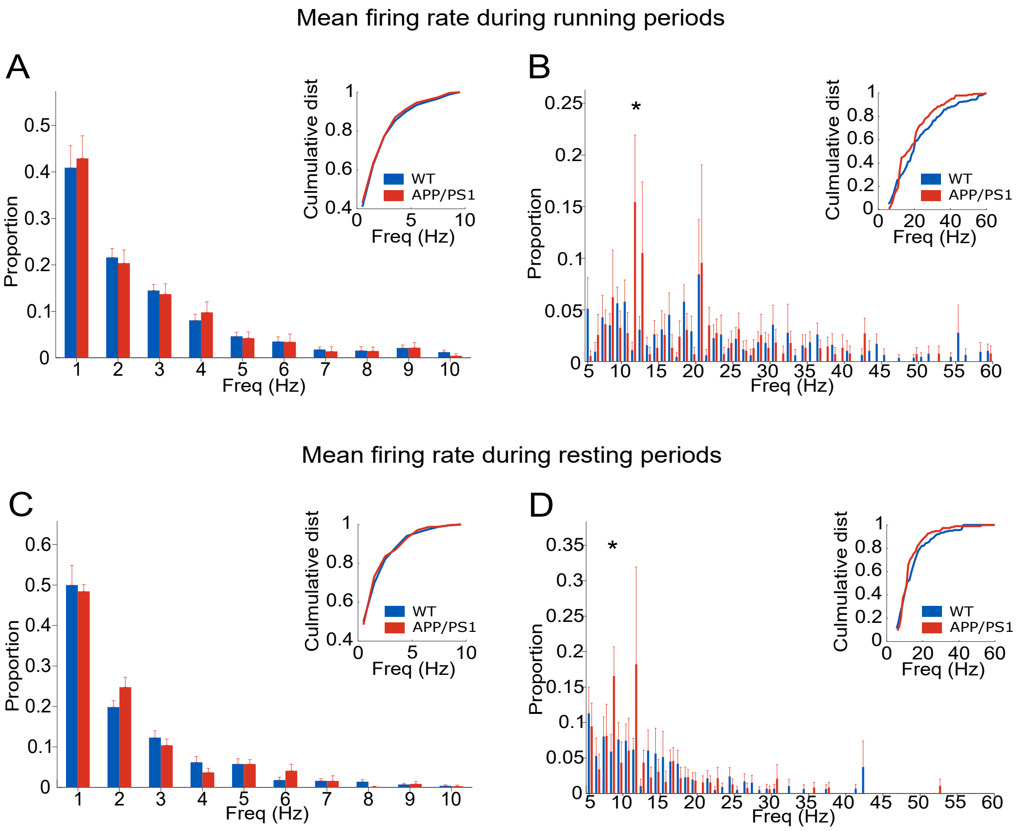

Supplementary Figure 4 Firing-rate distributions of hippocampal neurons during running and resting periods in awake young APP/PS1 mice

(A–B) Distribution of mean firing rates for putative pyramidal neurons (**A**) and putative fast-spiking interneurons (**B**) during running periods, analyzed per animal. Insets show the averaged cumulative distributions of firing frequencies for WT and APP/PS1 mice. Fast-spiking interneurons exhibited an altered distribution with a trend of increased fraction of low-frequency firing cells in APP/PS1 mice. * indicates frequency bin with significant genotype difference (t-test, P = 0.026 at 11 Hz). Insets show averaged cumulative distributions.

(C–D) Distribution of mean firing rates for putative pyramidal neurons **(C)** and putative fast-spiking interneurons **(D)** during resting periods, analyzed per animal. Insets show averaged cumulative distributions. APP/PS1 mice showed an altered distribution with the trend of an increased fraction of low-frequency firing fast-spiking interneurons. * indicates frequency bin with significant genotype difference (t-test, P = 0.036 at 8 Hz).

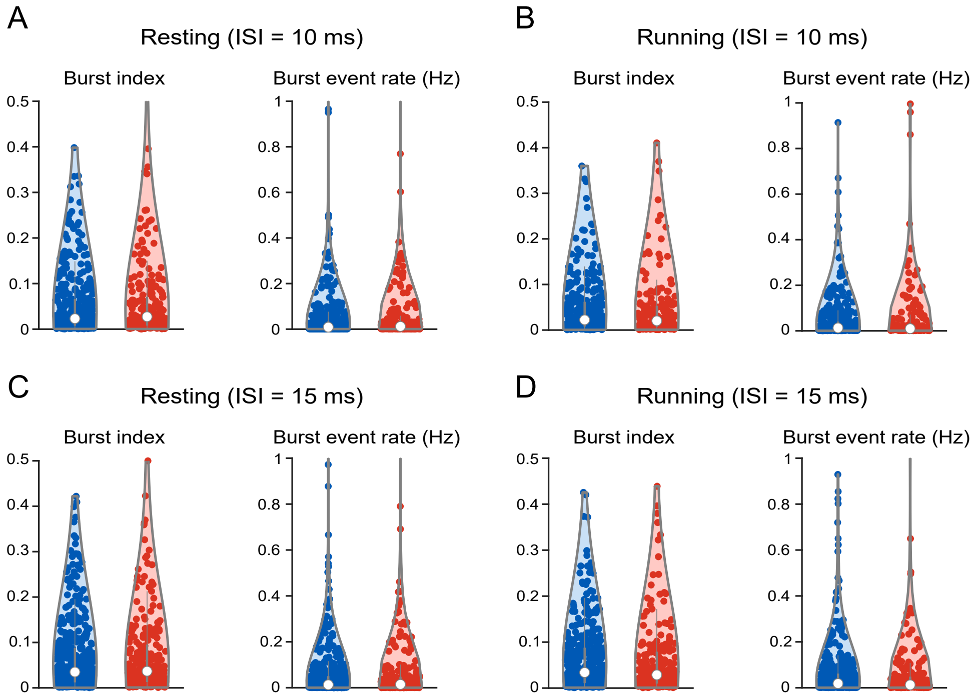

Supplementary Figure 5 Bursting activity in CA1 pyramidal cells analyzed using alternative ISI thresholds

(A) Resting (ISI = 10 ms). Left: burst index (WT = 0.050 ± 0.003, n = 414 (9 animals); APP/PS1 = 0.061 ± 0.006, n = 222 (7 animals); $P = 0.310$, $F_{(1,634)} = 1.031$). Right: burst event rate (WT = 0.043 ± 0.006 Hz; APP/PS1 = 0.082 ± 0.006 Hz; $P = 0.492$, $F_{(1,634)} = 0.472$).

(B) Running (ISI = 10 ms). Left: burst index (WT = 0.045 ± 0.003, n = 362 (9 animals); APP/PS1 = 0.045 ± 0.004, n = 194 (7 animals); $P = 0.791$, $F_{(1,554)} = 0.070$). Right: burst event rate (WT = 0.045 ± 0.005 Hz; APP/PS1 = 0.083 ± 0.031 Hz; $P = 0.907$, $F_{(1,554)} = 0.014$).

(C) Resting (ISI = 15 ms). Left: burst index (WT = 0.066 ± 0.004, n = 460 (9 animals); APP/PS1 = 0.075 ± 0.006, n = 255 (7 animals); P = 0.680, F(1,713) = 0.170). Right: burst event rate (WT = 0.056 ± 0.006 Hz; APP/PS1 = 0.087 ± 0.026 Hz; P = 0.978, $F_{(1,713)} = 0.001$).

(D) Running (ISI = 15 ms). Left: burst index (WT = 0.061 ± 0.003, n = 412 (9 animals); APP/PS1 = 0.057 ± 0.005, n = 222 (7 animals); $P = 0.786$, $F_{(1,632)} = 0.074$). Right: burst event rate (WT = 0.062 ± 0.006 Hz; APP/PS1 = 0.091 ± 0.030 Hz; $P = 0.870$, $F_{(1,632)} = 0.027$).

Data is represented as a single value per cell. WT in blue, APP/PS1 in red.

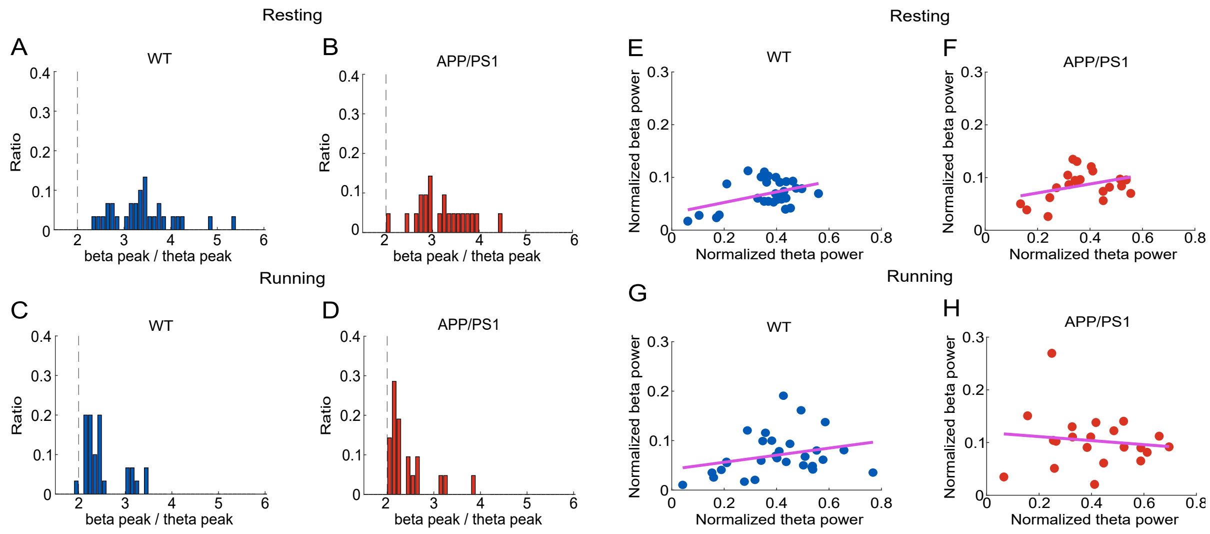

Supplementary Figure 6 Assessment of potential theta harmonic content in beta oscillations in awake mice

(A–D) Histograms show the distribution of beta peak/theta peak values, computed from the aperiodic-subtracted power spectrum for each recording session during resting (**A**, **B**) and running (**C**, **D**) in WT (**A**, **C**) and APP/PS1 mice (**B**, **D**). Theta range: 4–12 Hz; Beta range: 15–25 Hz. The vertical dashed line indicates the expected second harmonic of theta at the value of 2. Note sessions within 2 ± 0.15 were rare during resting (WT: 0/30; APP/PS1: 1/21) and remained a minority during running (WT: 4/30; APP/PS1: 6/21), with no significant genotype difference in both behavioral conditions in near-2 prevalence (two-sided Fisher's exact test: resting $P = 0.41$; running $P = 0.28$).

(E–H) Scatter plots show normalized beta-band (15–25 Hz) power versus theta-band (4–12 Hz) power, computed from aperiodic-subtracted power spectrum for each recording session during resting (**E**, **F**) and running (**G**, **H**) in WT (**E**, **G**) and APP/PS1 mice (**F**, **H**). Each dot represents one session, and the magenta line indicates a linear regression fit. No significant correlation was detected in either genotype or behavioral condition. Spearman correlation statistics are (**E**) WT, resting: $\rho = 0.21$, $P = 0.25$; (**F**) APP/PS1, resting: $\rho = 0.28$, $P = 0.21$; (**G**) WT, running: $\rho = 0.33$, $P = 0.07$; (**H**) APP/PS1, running: $\rho = -0.14$, $P = 0.55$.

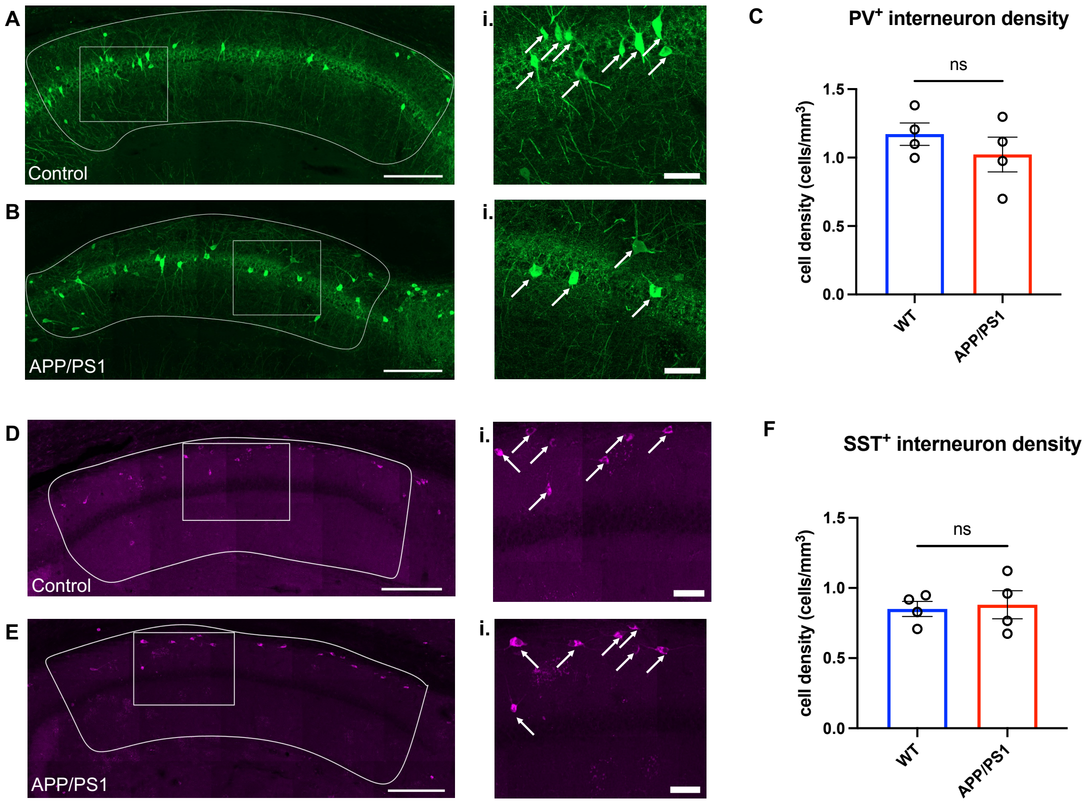

Supplementary Figure 7 Parvalbumin (PV)$^{+}$ and somatostatin (SST)$^{+}$ interneuron density in the hippocampal CA1 region of young mice

(A–B) Representative images showing immunofluorescence staining of PV^{+} interneurons in **A.** control and **B.** APP/PS1 hippocampi of young mice. Scale bar: 200 μm. Manually surrounded area represents CA1 hippocampal area used for PV^{+} interneuron density quantification. **i.** Enlarged representative image of the CA1 area of the hippocampus of the boxed areas in A and B. Arrows indicate positively stained neurons. Scale bar: 50 μm.

(C) Data showing cell density for PV^{+} interneurons in the hippocampal CA1 region of young mice. Comparisons were done with an unpaired t-test. Error bars represent mean ± SEM, ns: not significant.

(D–E) Representative images showing immunofluorescence staining of SST^{+} interneurons in **D.** control and **E.** APP/PS1 hippocampi of young mice. Scale bar: 200 μm. Manually surrounded area represents the CA1 hippocampal area used for PV interneuron density quantification. **i.** Enlarged representative image of the CA1 area of the hippocampus of the boxed areas in A and B. Arrows indicate positively stained neurons. Scale bar: 50 μm.

(F) Data showing cell density for SST^{+} interneurons in the hippocampal CA1 region of young mice. Comparisons were done with an unpaired t-test. Error bars represent mean ± SEM, ns: not significant.

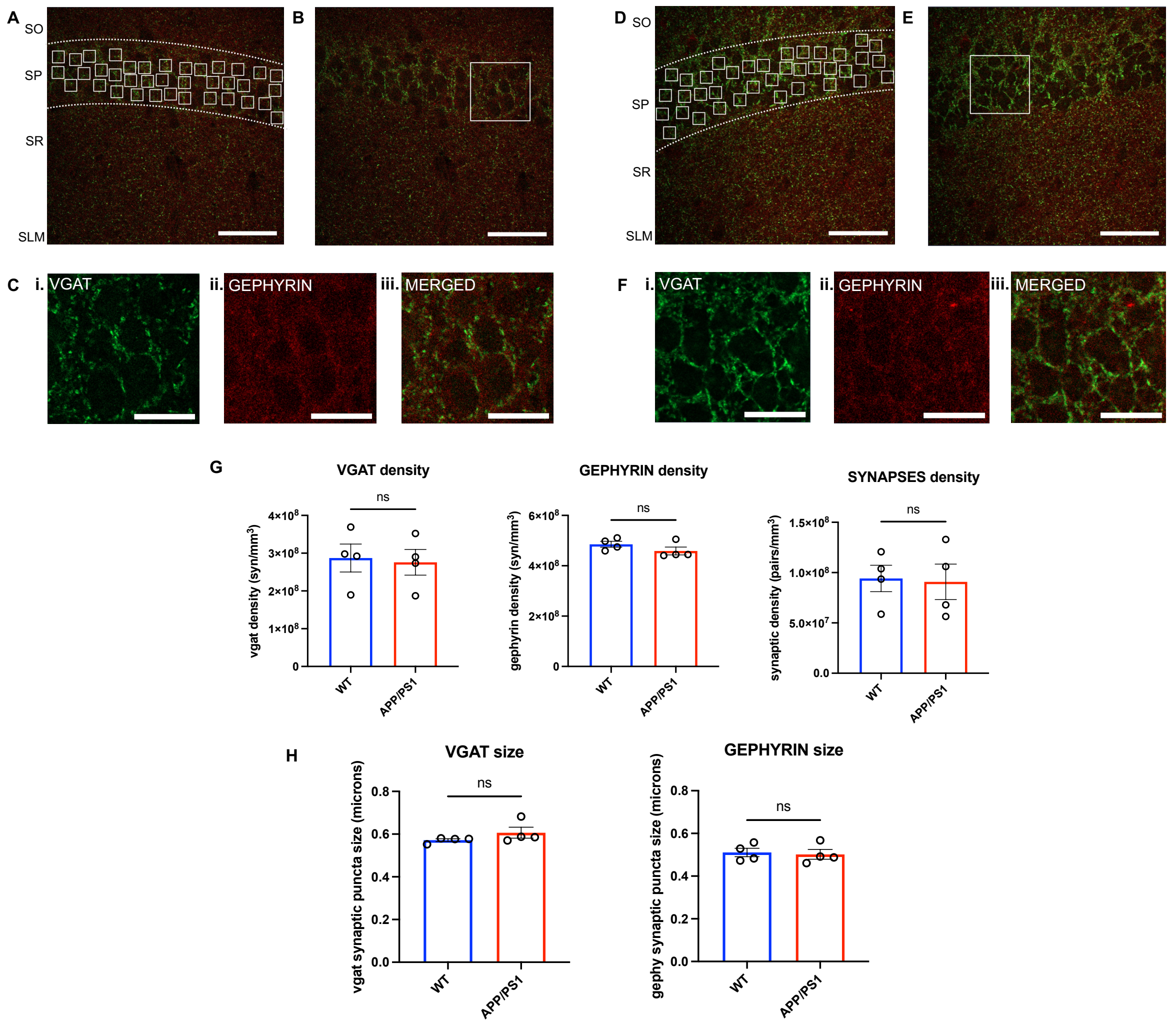

Supplementary Figure 8 Quantification of perisomatic inhibitory synapses in the hippocampal CA1 region of young mice

(A) Representative immunostaining of VGAT and gephyrin expression in the hippocampal CA1 of young WT mouse, with white regions of interest (ROIs) (10x10 μm) indicating regions used for perisomatic inhibitory synapse quantification. Scale bar: 50 μm.

(B–C) Enlarged view of boxed area in panel B showing **i.** VGAT stain (perisomatic basket shape pattern observed), **ii.** Gephyrin stain and **iii.** synaptic pairs (VGAT and gephyrin merged). Scale bar: 20 μm.

(**D)** Representative immunostaining of VGAT and gephyrin expression in the hippocampal CA1 of young APP/PS1 mice, with white regions of interest (ROIs) (10x10μm) indicating regions used for perisomatic inhibitory synapse quantification. Scale bar: 50 μm.

(**E–F)** Enlarged view of boxed area in panel E showing **i.** VGAT stain (perisomatic basket shape pattern observed), **ii.** Gephyrin stain and **iii.** synaptic pairs (VGAT and gephyrin merged). Scale bar: 20 μm.

(**G**) Data showing quantification of VGAT, gephyrin (synapses/mm^3), and synaptic pairs density (pairs/mm^3), respectively. The brain region quantified was the pyramidal layer of the hippocampal layer CA1. Statistical analysis was done using an unpaired t-test. Error bars represent mean ± SEM, ns: non-significant.

(**H**) Data showing synaptic puncta size in microns for VGAT and gephyrin synapses, respectively. The brain region quantified was the pyramidal layer of the hippocampal layer CA1. Statistical analysis was done using an unpaired t-test. Error bars represent mean ± SEM, ns: non-significant.

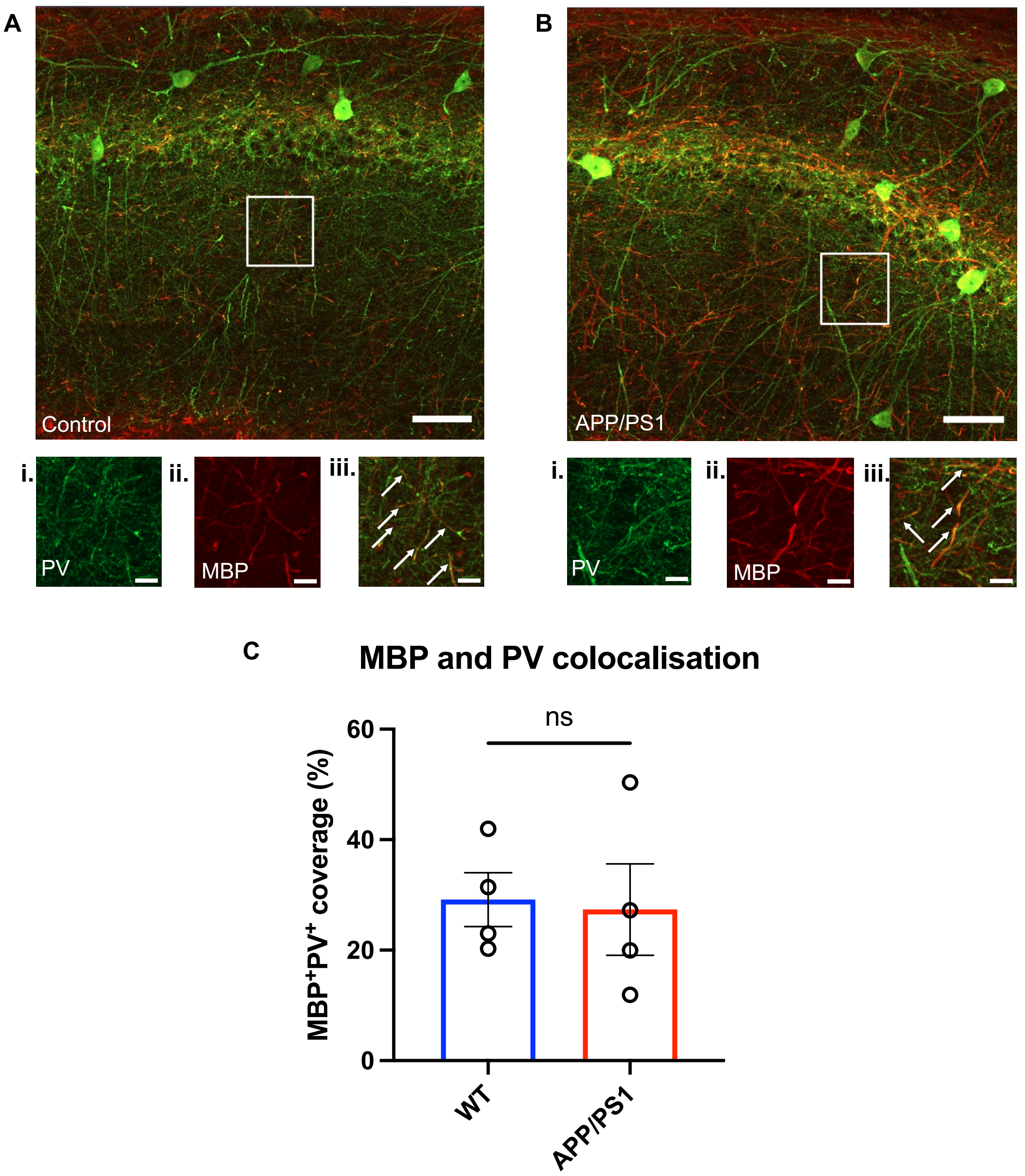

Supplementary Figure 9 Myelination of the hippocampal CA1 parvalbumin-positive interneurons in young mice

(A–B) Representative images of WT (**A**) and APP/PS1 (**B**) CA1 region showing immunoreactivity for parvalbumin (PV) and myelin basic protein (MBP) in young mice. Scale bar: 50 µm. Inset images of the frame areas in A and B show single channels of **i**. PV and **ii**. MBP and **iii**. merged

channels. Arrows in iii. indicate overlap of PV and MBP immunoreactivity. Scale bar represents 10 μm

(**C**) Data showing relative colocalization of MBP and PV in the CA1 of young WT and APP/PS1 mice. The PV$^+$ and MBP$^+$ area was expressed as a percentage of the total PV$^+$ area. Statistical analysis was done with an unpaired t-test. Error bars represent mean ± SEM, ns: not significant.

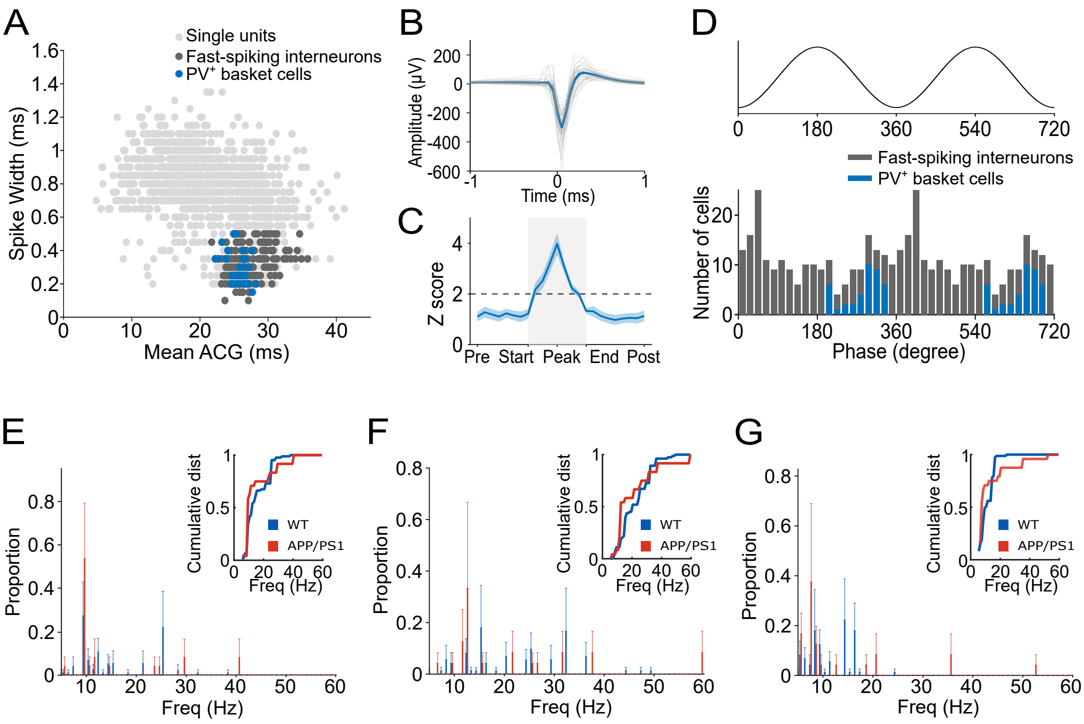

Supplementary Figure 10 Identification and firing properties of putative PV$^+$ basket cells in young WT and APP/PS1 mice

(A) Classification of putative PV$^+$ basket interneurons (blue) based on spike width and the first moment of the autocorrelogram compared with all recorded single units (gray).

(B) Average spike waveform of putative PV$^+$ basket cell (thick line = mean ± SEM; thin lines = individual units).

(C) Ripple-triggered average firing of putative PV$^+$ basket cells showing significant modulation around the peak of ripple events (Z-score).

(D) Preferred theta-phase distribution of PV$^+$ basket cells (blue) compared with other fast-spiking interneurons (gray), aligned to the schematic theta oscillation (top).

(E–G) Mean firing-rate distributions of putative PV$^+$ basket cells, 26 cells from 9 WT animals, and 14 cells from 7 APP/PS1 animals. **E**: Mean firing rate across the entire recording session. **F**: Mean firing rate during running periods. **G**: Mean firing rate during resting periods. Insets show cumulative distributions comparing WT (blue) and APP/PS1 (red) mice. No significant differences in firing rates were observed between genotypes.

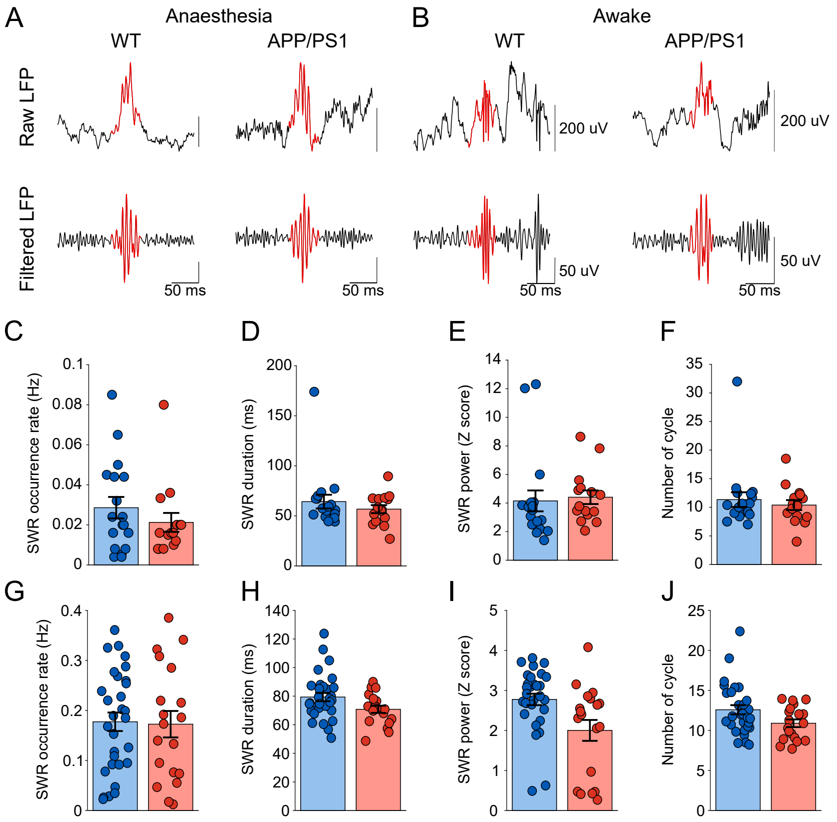

Supplementary Figure 11 Properties of sharp wave ripples in young APP/PS1 mice *in vivo*

(A–B) Example CA1 LFP traces showing spontaneous sharp wave ripple (SWR) events recorded *in vivo* under anesthesia **(A)** and in awake condition during resting **(B)** in WT and APP/PS1 mice. Top: raw LFP with detected SWRs highlighted in red. Bottom: corresponding ripple-band-filtered traces (100–250 Hz).

(C) SWR occurrence rate under anesthesia. No group difference (WT = 0.029 ± 0.006 Hz, APP/PS1 = 0.021 ± 0.005 Hz; P = 0.361, $F_{(1,31)}$ = 0.859).

(D) SWR duration during anesthesia. WT and APP/PS1 mice were comparable (WT = 64 ± 7 ms, APP/PS1 = 57 ± 4 ms; *P* = 0.773, $F_{(1,31)}$ = 0.085).

(E) SWR power under anesthesia. No group effect (WT = 4.151 ± 0.731, APP/PS1 = 4.409 ± 0.477; P = 0.548, $F_{(1,31)}$ = 0.369).

(F) Ripple cycle number under anesthesia. No significant difference (WT = 11.343 ± 1.289, APP/PS1 = 10.389 ± 0.867; *P* = 0.308, $F_{(1,31)}$ = 1.073).

(G) SWR occurrence rate during resting in awake recordings. Groups did not differ (WT = 0.177 ± 0.019 Hz, APP/PS1 = 0.173 ± 0.026 Hz; P = 0.776, $F_{(1,47)}$ = 0.082).

(H) SWR duration during resting in awake recordings. No genotype effect (WT = 0.079 ± 0.003, APP/PS1 = 0.071 ± 0.003; P = 0.178, $F_{(1,47)}$ = 1.879).

(I) SWR power during resting in awake recordings. A trend toward reduced power in APP/PS1 mice (WT = 2.776 ± 0.143, APP/PS1 = 2.001 ± 0.261; P = 0.059, $F_{(1,47)}$ = 3.756).

(J) Ripple cycle number during resting in awake recordings. No significant difference (WT = 12.586 ± 0.574, APP/PS1 = 10.898 ± 0.481; P = 0.175, $F_{(1,47)}$ = 1.897).

Data are presented as mean ± SEM (anesthesia: WT n = 19 animals, APP/PS1 n = 15 animals; awake: WT n = 30 recordings (9 animals), APP/PS1 n = 21 recordings (7 animals)). WT in blue, APP/PS1 in red.
