# Supplementary material for: Reduced inhibition, bursting, and accelerated oscillations drive early hippocampal hyperactivity in Alzheimer’s disease in vivo
Source: Commun Biol. 2026 Mar 28;9:688. doi: 10.1038/s42003-026-09918-y (PMC13195132; doi:10.1038/s42003-026-09918-y)
Supplement: Supplementary file 2 — Description of Additional Supplementary Files [file 42003_2026_9918_MOESM2_ESM.docx]

**Description of Additional Supplementary File**

File name: Supplementary Data
Description: Statistical details for each figure and supplementary figure in the paper.
