# Supplementary material for: Reduced inhibition, bursting, and accelerated oscillations drive early hippocampal hyperactivity in Alzheimer’s disease in vivo
Source: Commun Biol. 2026 Mar 28;9:688. doi: 10.1038/s42003-026-09918-y (PMC13195132; doi:10.1038/s42003-026-09918-y)
Supplement: Supplementary file 4 — Reporting Summary [file 42003_2026_9918_MOESM4_ESM.pdf]

nature portfolio

Corresponding author(s): Jian Gan

Last updated by author(s): Mar 4, 2026

Reporting Summary

Nature Portfolio wishes to improve the reproducibility of the work that we publish. This form provides structure for consistency and transparency in reporting. For further information on Nature Portfolio policies, see our Editorial Policies and the Editorial Policy Checklist.

Statistics

For all statistical analyses, confirm that the following items are present in the figure legend, table legend, main text, or Methods section.

n/a	Confirmed	
☐	☒	The exact sample size (*n*) for each experimental group/condition, given as a discrete number and unit of measurement
☐	☒	A statement on whether measurements were taken from distinct samples or whether the same sample was measured repeatedly
☐	☒	The statistical test(s) used AND whether they are one- or two-sided<br>*Only common tests should be described solely by name; describe more complex techniques in the Methods section.*
☐	☒	A description of all covariates tested
☐	☒	A description of any assumptions or corrections, such as tests of normality and adjustment for multiple comparisons
☐	☒	A full description of the statistical parameters including central tendency (e.g. means) or other basic estimates (e.g. regression coefficient) AND variation (e.g. standard deviation) or associated estimates of uncertainty (e.g. confidence intervals)
☐	☒	For null hypothesis testing, the test statistic (e.g. *F*, *t*, *r*) with confidence intervals, effect sizes, degrees of freedom and *P* value noted<br>*Give P values as exact values whenever suitable.*
☒	☐	For Bayesian analysis, information on the choice of priors and Markov chain Monte Carlo settings
☒	☐	For hierarchical and complex designs, identification of the appropriate level for tests and full reporting of outcomes
☐	☒	Estimates of effect sizes (e.g. Cohen's *d*, Pearson's *r*), indicating how they were calculated

Our web collection on statistics for biologists contains articles on many of the points above.

Software and code

Policy information about availability of computer code

Data collection	Patchmaster v2x91for patch-clamp and LFP recordings. Intan RHX data acquisition software and PhenoSys recording software for probe recordings. ZEN Microscopy Software, ImageJ and Fiji software, and Matlab for immunostainings
Data analysis	Matlab R2024b; Kilosort 3; Phy 2; ZEN Microscopy Software; ImageJ and Fiji; GraphPad Prism 10.3

For manuscripts utilizing custom algorithms or software that are central to the research but not yet described in published literature, software must be made available to editors and reviewers. We strongly encourage code deposition in a community repository (e.g. GitHub). See the Nature Portfolio guidelines for submitting code & software for further information.

Data

Policy information about availability of data

All manuscripts must include a data availability statement. This statement should provide the following information, where applicable:

- Accession codes, unique identifiers, or web links for publicly available datasets
- A description of any restrictions on data availability
- For clinical datasets or third party data, please ensure that the statement adheres to our policy

Numerical source data for graphs and quantitative comparisons presented in this paper are deposited at Mendeley Data: DOI: 10.17632/dpy4jt7d7v.1. Any additional information required to reanalyze the data will be available from the corresponding author on reasonable request.

Research involving human participants, their data, or biological material

Policy information about studies with human participants or human data. See also policy information about sex, gender (identity/presentation), and sexual orientation and race, ethnicity and racism.

Reporting on sex and gender	N/A
Reporting on race, ethnicity, or other socially relevant groupings	N/A
Population characteristics	N/A
Recruitment	N/A
Ethics oversight	N/A

Note that full information on the approval of the study protocol must also be provided in the manuscript.

Field-specific reporting

Please select the one below that is the best fit for your research. If you are not sure, read the appropriate sections before making your selection.

☒ Life sciences ☐ Behavioural & social sciences ☐ Ecological, evolutionary & environmental sciences

For a reference copy of the document with all sections, see nature.com/documents/nr-reporting-summary-flat.pdf

Life sciences study design

All studies must disclose on these points even when the disclosure is negative.

Sample size	No sample size calculation was performed. Sample size was chosen according to authors' previous papers. The sample size used in the paper was comparable with those commonly used in the field of neurophysiology and systems neuroscience.
Data exclusions	We did not exclude data.
Replication	Technical replicates as well as biological replicates are clearly mentioned in the manuscript. The number of replicates was comparable with commonly used in the field of neurophysiology and systems neuroscience.
Randomization	Animals were randomly selected.
Blinding	Genotype results were blind to experimenters during data collection and analysis.

Reporting for specific materials, systems and methods

We require information from authors about some types of materials, experimental systems and methods used in many studies. Here, indicate whether each material, system or method listed is relevant to your study. If you are not sure if a list item applies to your research, read the appropriate section before selecting a response.

Materials & experimental systems

n/a	Involved in the study	
☐	☒	Antibodies
☒	☐	Eukaryotic cell lines
☒	☐	Palaeontology and archaeology
☐	☒	Animals and other organisms
☒	☐	Clinical data
☒	☐	Dual use research of concern
☒	☐	Plants

Methods

n/a	Involved in the study	
☒	☐	ChIP-seq
☒	☐	Flow cytometry
☒	☐	MRI-based neuroimaging

Antibodies

Antibodies used	parvalbumin (rabbit,PV27a, 1:1000, Swant), somatostatin (rabbit, T-4103, 1:1000, BMA Biomedicals), VGAT (guinea pig, 131004, 1:250, Synaptic Systems), gephyrin (mouse, 147021, 1:100, Synaptic Systems), MBP (rat, MCA4095, 1:300, BioRad), β-amyloid 6E10 (mouse, 803004, 1:1000, BioLegend). The secondary antibodies were as follows: Alexa Fluor 594 goat anti-mouse (A11005, 1:1000,

	Thermo Fisher Scientific), Alexa Fluor 488 goat antiguinea pig (A11073, 1:1000, Thermo Fisher Scientific), Alexa Fluor 594 goat anti-rat (A11007, 1:1000, Thermo Fisher Scientific), Alexa Fluor 488 goat anti-mouse (A21121, 1:1000, Thermo Fisher Scientific), Alexa Fluor 488 goat anti-rabbit (A11008, 1:500, Thermo Fisher Scientific).
Validation	We validated the antibodies used above. Biological replicates are indicated in the figures and figure legends.

Animals and other research organisms

Policy information about studies involving animals; ARRIVE guidelines recommended for reporting animal research, and Sex and Gender in Research

Laboratory animals	APPswe/PS1ΔE9 mice, C57BL/6J background. 2- to 3-month-old for patch-clamp and LFP recordings. 3- to 5-month-old for probe recordings
Wild animals	No
Reporting on sex	Both male and female mice were used. We did not perform a sex-based analysis.
Field-collected samples	No
Ethics oversight	UK Home Office project licence (PP8564759). University of Edinburgh Animal Welfare and Ethical Review Body

Note that full information on the approval of the study protocol must also be provided in the manuscript.

Plants

Seed stocks	N/A
Novel plant genotypes	N/A
Authentication	N/A
